# Ionic Bent-Core Pillar[*n*]arenes: From Liquid Crystals to Nanoaggregates and Functional Applications

**DOI:** 10.1021/acs.chemmater.4c01952

**Published:** 2024-10-02

**Authors:** Iván Marín, Martín Castillo-Vallés, Rosa I. Merino, César L. Folcia, Joaquín Barberá, M. Blanca Ros, José L. Serrano

**Affiliations:** † Instituto de Nanociencia y Materiales de Aragón (INMA), CSIC-Universidad de Zaragoza, 50009 Zaragoza, Spain; ‡ Departamento de Química Orgánica, Facultad de Ciencias, Universidad de Zaragoza, 50009 Zaragoza, Spain; § Departamento de Física de la Materia Condensada, Facultad de Ciencias, CSIC-Universidad de Zaragoza, 50009 Zaragoza, Spain; ∥ Departamento de Física, Facultad de Ciencia y Tecnología, Universidad del País Vasco, E-48080 Bilbao, Spain

## Abstract

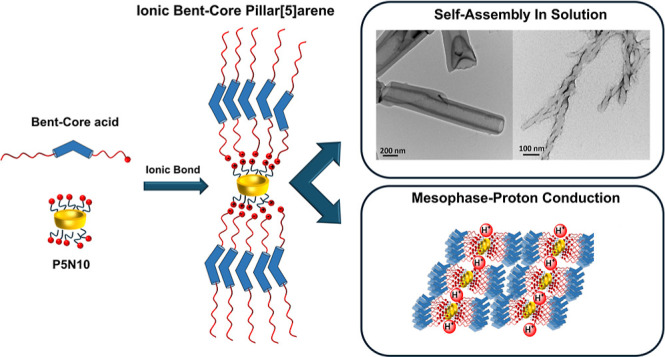

Herein, we report the first examples of supramolecular systems from bent-core-based pillar[*n*]arenes through ionic bonds. These ionic materials have been prepared by the interaction of an amino-ended pillar[5]arene (P5N10) and three different carboxylic acids, including bent-core moieties. The bent-core units are based on ester, biphenyl, and azobenzene structures bearing two different flexible spacers between the carboxyl group and the central bent-core aromatic units. The ionic pairs segregate the molecular blocks, leading to columnar liquid crystal organizations. These ionic supramolecular compounds exhibit interesting results as proton-conductive materials. Furthermore, the introduction of azobenzene units in the bent-core structure has provided a photoresponse to the proton conduction materials. Interestingly, the amphiphilic character generated by the ionic pairs and the hydrophobic bent-core structures allows their molecular self-assembly in water solution, resulting in aggregates of appealing morphologies. The structural modifications of the bent-core units (i.e., connecting bonds at the lateral structure and spacer lengths) provide an attractive analysis on the relationship between the chemical structure and the morphology of the aggregates (fibers, chiral ribbons, nanotubes...). Additionally, the self-assembly process and evolution of the aggregates from fibers to nanotubes have been studied with several techniques.

## 1 Introduction

Since the discovery of pillar[*n*]arenes in 2008,^1^ these macrocycles have undergone a thoughtful impact in the chemical society due to their unique properties such as electron-rich cavity,^2^ symmetrical pillar-shaped frame,^3^ planar chirality,^4^ or controllable cavity size.^5,6^ These properties have led a breakthrough in supramolecular chemistry with the application of pillar[*n*]arenes in a wide number of fields including drug delivery,^7,8^ fluorescent materials,^9,10^ metal recognition,^11^ chiral assemblies,^12,13^ or separation materials.^14,15^ In the route to advanced functional materials, the liquid crystal (LC) state has proved to play a very interesting and versatile role when dimensionality, processing, or mobility is aimed,^16,17^ including classical but also nonconventional molecular designs.^18,19^ However, the number of mesomorphic pillar[*n*]arenes is so far limited,^20−22^ probably due to synthetic difficulties. In most of the examples described, promesogenic units are introduced in the symmetric structure of the pillar[*n*]arenes taking use of “click” reactions.^21,22^ Alternatively, an innovative and easy approach to prepare pillar[*n*]arene-based LCs was described recently by our group^23^ by using ionic bonding. This noncovalent approach is guided by electrostatic interactions that allow molecular self-organization either in bulk, even without the presence of mesogenic units, or in the presence of liquids,^24,25^ being a very attractive alternative to structure-self-assembly processes. Besides avoiding time-consuming synthesis, this synthetic method^26^ provides materials with applicability from proton conduction in bulk to a variety of nanostructured aggregates in water suitable for encapsulation targets.

In the last two decades, ionic LCs have become of great interest as they combine properties of LCs and ionic liquids, exploring for them hot-topic applications. In this target, mainly calamitic and discotic mesophases have been explored,^27−30^ while ionic bent-core liquid crystals are scarce^31−33^ in spite of the outstanding novelties provided by the novel bent-core mesophases.^25,34^ These pointed properties stand out for the tendency of their bent molecules to self-assemble in compact and polar organizations.^35−40^ These structural characteristics are transmitted to the bulk material with unique polar and chiral mesophases [SmCP, helical nanofilament mesophases (HNFs), HLNCs...] and functional possibilities.^31,41−44^ But alternatively, these bent-core molecules have also evidenced an outstanding supramolecular versatility either in solution, leading from fibers to helical ribbons and nanotubes or to gels, or onto surfaces, promoting LB and SAM thin films,^45,46^ providing them very attractive possibilities in the soft-materials application fields.

**Figure 1:**
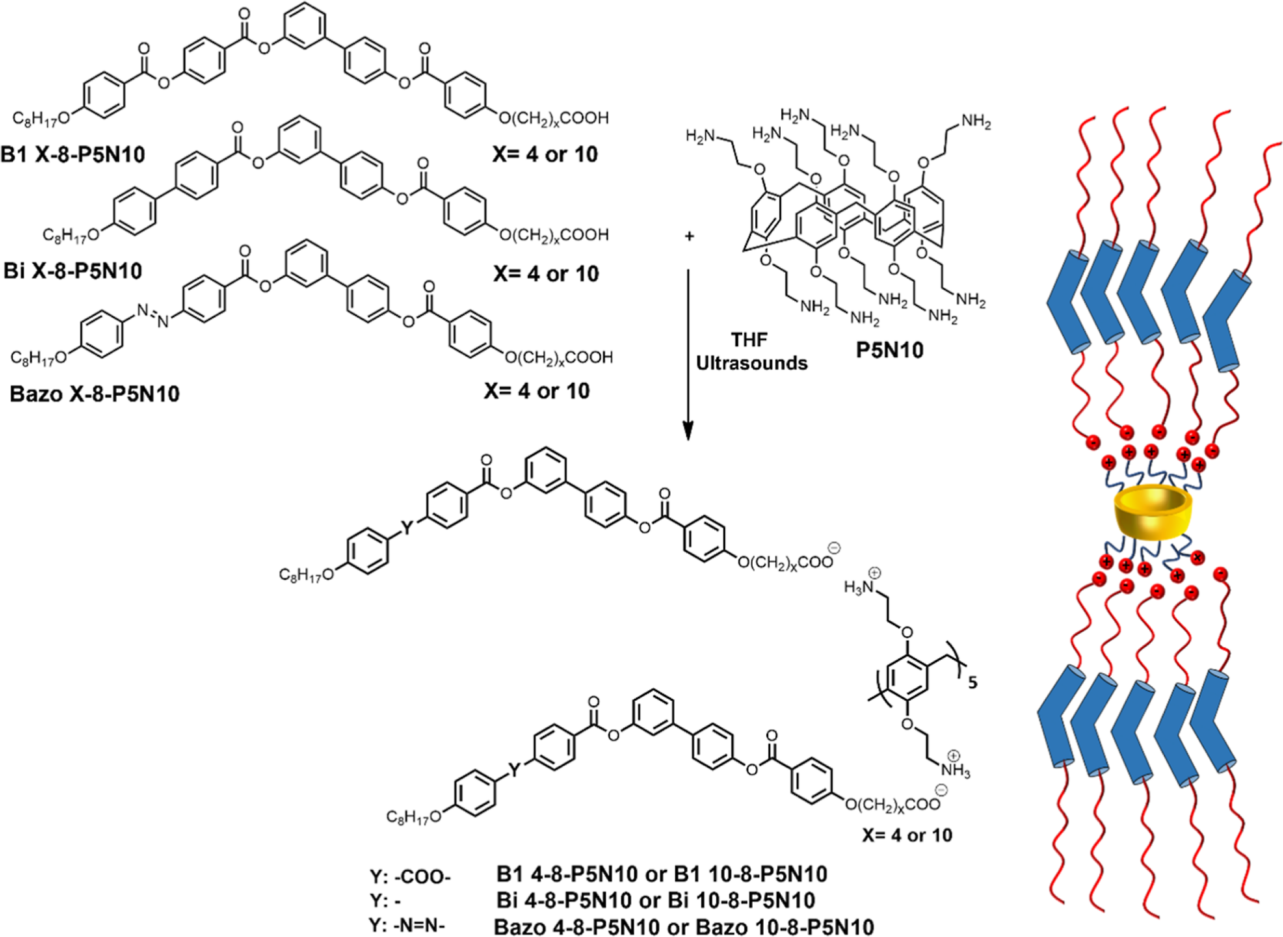
Chemical structures of BC and pillar[5]arene components, synthesis of the ionic superstructures, and schematic representation of the ionic BC pillar[5]arenes.

Herein, we report an unprecedented synergistic approach for pillar[*n*]arenes and bent-core moieties, describing the first examples of ionic bent-core pillar[*n*]arenes and their supramolecular potential, leading to both liquid crystal behavior and water self-assembly and exploring their applicability in several fields. The ionic materials have been prepared through an easy and fast method, mixing a pillar[5]arene (**P5N10**) that contains ten terminal amine groups and different bent-core (BC) carboxylic acids. According to our previous results^31,33^ and to explore functional possibilities, a selection of BC structures, labeled as **BC X-8-P5N10**, have been considered (Figure 1). They consist of a well-defined asymmetric structure bearing a 5-ring BC structure. An 8-carbon atom aliphatic chain is incorporated at one end, and a flexible tail with 4 or 10 (x) carbon atoms is grafted at the other, ending with the –COOH group. Three different connections have been considered for the lateral cores of the BC structures: a common ester linking group (B1) broadly used in our previous studies with strong tendency for BC mesophases^31^ (compounds **B1 X-8-P5N10**); the straight union via a biphenyl (Bi) moiety, inspired by the trend of biphenyl lateral cores to promote the HNF mesophase formation^31,47,51^ (compounds **Bi X-8-P5N10**); and an azo linker (Bazo) (compounds **Bazo X-8-P5N10**) in order to obtain photoresponsive materials.^33,48^ The ionic pillar[5]arene-based compounds promote self-organized liquid crystal materials although nonmesogenic BC units have been used, yielding oblique columnar and rectangular columnar mesophases; moreover, in condensed phases, these ionic materials have been explored with good proton conduction properties. The introduction of azobenzenes in the BC structure has provided a photoresponse to the proton conduction materials. Furthermore, the ionic moieties, which provide a hydrophilic character to these molecules, turned them into very attractive amphiphilic materials. Thus, ionic BC pillar[5]arenes self-assembled in aqueous media, leading to a wide variety of nanostructures, with different morphologies depending on both bond (Y) on the lateral structure and the spacer length (*x*) on the BC moiety.

## 2 Results and Discussion

### 2.1 Preparation of Ionic Bent-Core-Based Pillar[5]arenes

The materials and techniques used, as well as the synthesis and characterization of the compounds prepared, are included in Supporting Information Sections 1–3. The BC acids employed in the synthesis of the ionic pillar[5]arenes differed in the aromatic moiety which set up the lateral structure, being phenyl benzoate (B1), biphenyl (Bi), and azobenzene (Bazo) derivatives with a terminal alkyl chain of 8 carbons in all cases. As revealed in our previous studies,^31−33^ the spacer length between the carboxyl group and the BC structure has demonstrated to be key in self-assembly processes. Consequently, we wanted to evaluate the influence of the flexible spacer in the BC pillar[5]arenes by introducing a short spacer (*x*:4) and a long spacer (*x*:10). The synthesis of the carboxylic acids,^31,32^**B1 4-8**, **B1 10-8**, **Bi 4-8**, **Bi 10-8**, **Bazo 4-8**, **Bazo 10-8**, and pillar[5]arene **P5N10**,^49^ was carried out following previously described procedures, keeping similar labels for the former. The ionic BC pillar[5]arenes were prepared by dissolving the corresponding carboxylic-functionalized BC derivatives and the polyamine compound **P5N10** in a ratio of 10:1 in THF (Figure 1). Then, the solution was ultrasonicated for 1 h to ensure the proton migration, the solvent evaporated, and the crude dried at 40 °C under vacuum until the weight remained stable. The H-transfer from the carboxylic acid to the amine groups resulting in the formation of the ionic pairs was corroborated by infrared spectroscopy (FT-IR) and nuclear magnetic resonance (NMR).

**Figure 2:**
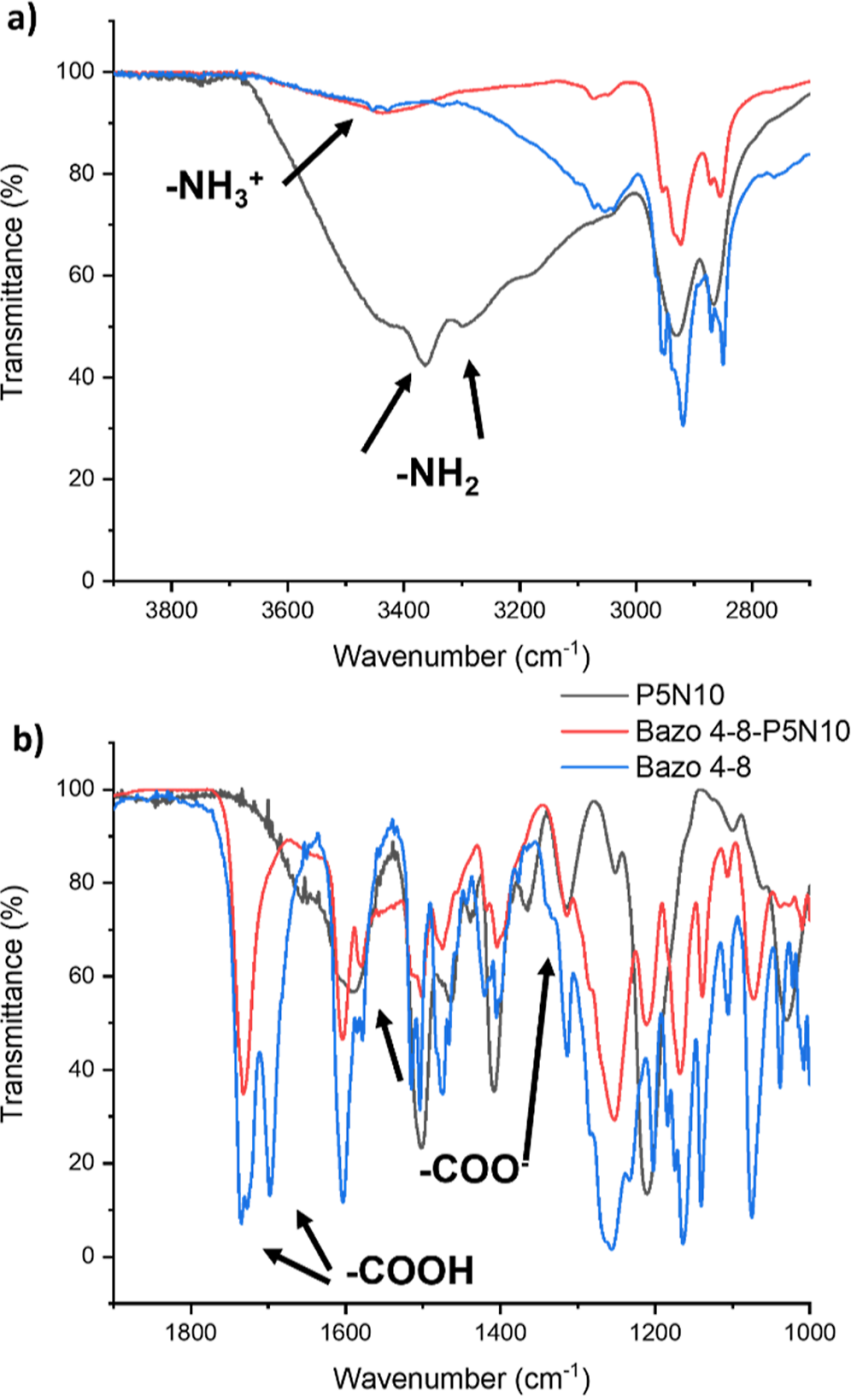
Representative and comparative FT-IR spectra of compounds **P5N10**, **Bazo 4-8-P5N10**, and **Bazo 4-8** (neat in KBr): (a) amine region and (b) carbonyl region.

In Figure 2, the FT-IR spectra of **P5N10**, **Bazo 4-8-P5N10**, and **Bazo 4-8** are compared as a representative prove of the ionic salt’s formation. In the amine region (Figure 2a), the stretching absorption produced by the pillar[5]arene amine groups (black line) disappeared, indicating proton transfer from the carboxylic acid groups to the amine moieties and the formation of the corresponding salt (−NH_3_^+^ –OCO−). Regarding the carbonyl zone (–C=O) (Figure 2b), BC-acid **Bazo 4-8** showed two bands from the dimeric (1697 cm^–1^) and free (1734 cm^–1^) forms of the carboxylic acid units. However, in the ionic complexes, the dimeric band disappeared, and two new absorptions appeared because of the symmetric and asymmetric carboxylate group vibrations 1580 and 1314 cm^–1^.

**Figure 3:**
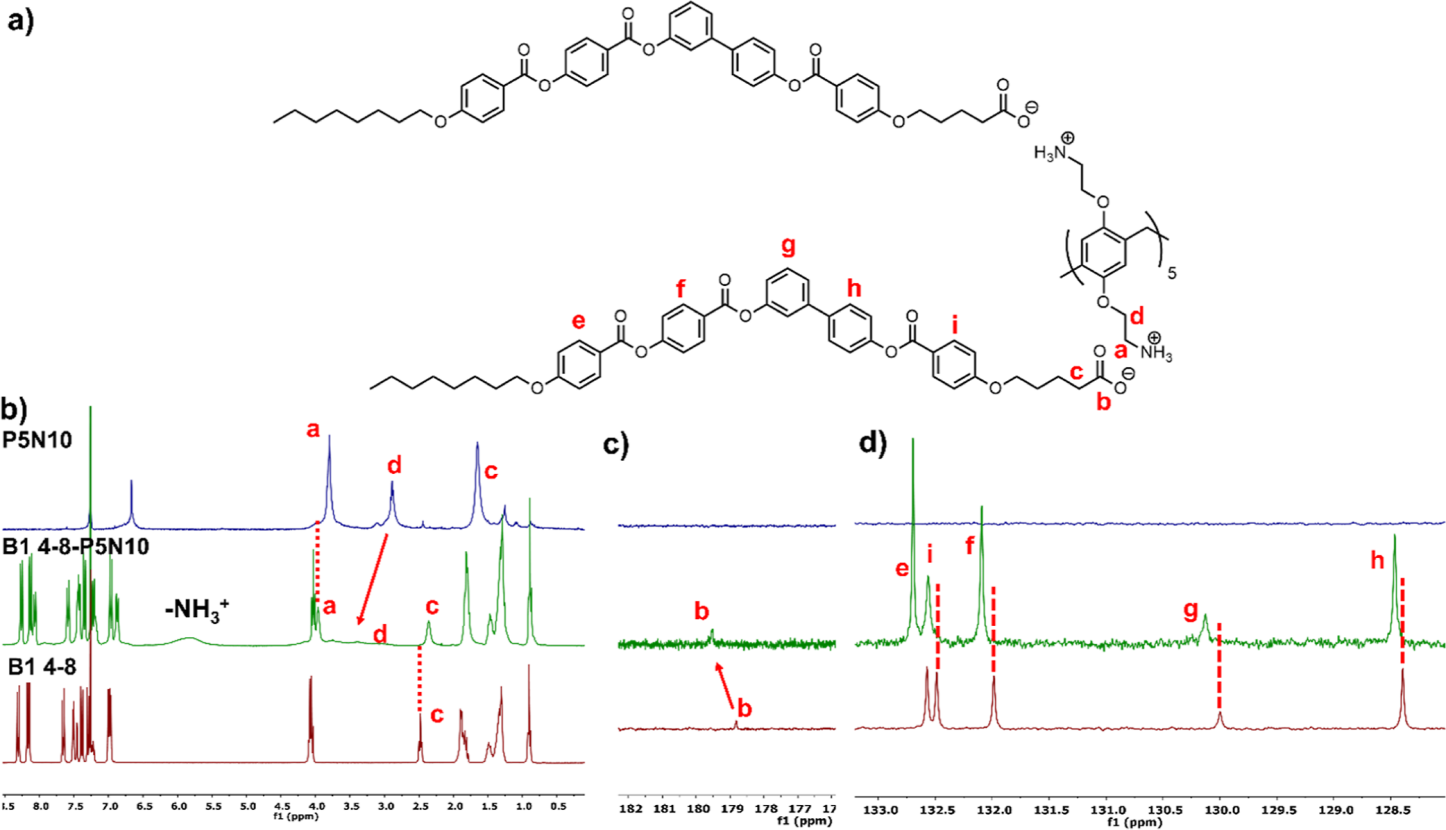
NMR comparative studies in CDCl_3_ solution: (a) **B1 4-8-P5N10** molecule with the signals affected by the ionic bond formation labeled in red, (b) ^1^H NMR spectra, and (c,d) ^13^C NMR spectra of **P5N10**, **B1 4-8-P5N10**, and **B1 4-8**.

The BC pillar[5]arenes were also studied by different NMR techniques: ^1^H NMR, ^13^C NMR, ^1^H–^1^H COSY, ^1^H–^13^C HSQC, and ^1^H–^13^C HMBC. These experiments were carried out in CDCl_3_ and showed unequivocally the formation of the ionic BC pillar[5]arene compounds (Figure 3). Thus, Figure 3b compares the ^1^H NMR spectra of **B1 4-8**, **P5N10**, and **B1 4-8-P5N10** showing the formation of the ionic bond as a broad band that appeared at 5.75 ppm for **B1 4-8-P5N10** due to the presence of the ammonium salt (−NH_3_^+^). Moreover, conclusive shifts of peaks corresponding to both components, carboxylic acids, and the pillar[5]arene **P5N10** were observed. These changes mainly affected protons located next to both–CO– of the BC moiety and the terminal N-containing unit of the pillar[5]arene block: –CH_2_COO– (*H*_c_) was shifted upfield from 2.48 to 2.36 ppm, –CH_2_NH_3_^+^ (*H*_a_) downfield from 2.88 to 3.38 ppm, and –OCH_2_CH_2_NH_3_^+^ (*H*_d_) was shifted from 3.80 to 3.96 ppm. The H-aromatic signals of the BC acid suffered small displacements attributed to π–π interaction between the aromatic rings of neighboring BC units favored by a characteristic compact packing even in solution.^31^

Regarding ^13^C NMR, several displacements confirmed the formation of the ionic bond: the –C=O– group resonance (*C*_b_) shifted from 178.80 to 179.60 ppm (Figure 3c). Moreover, in good agreement with ^1^H NMR spectral comments, peaks corresponding to *C*_g_, *C*_h_, *C*_f_, and *C*_i_ of the rigid BC showed noticeable upfield shifts in the ^13^C NMR spectra, consistent with the interaction between the BC moieties (Figure 3d, see Supporting Information Section 3.1 for further information).

### 2.2 Liquid Crystalline Properties

The thermal properties and mesomorphic character of the ionic complexes were analyzed by polarized optical microscopy (POM), thermogravimetry analysis (TGA), differential scanning calorimetry (DSC), and X-ray diffraction (XRD) at variable temperature.

**Figure 4:**
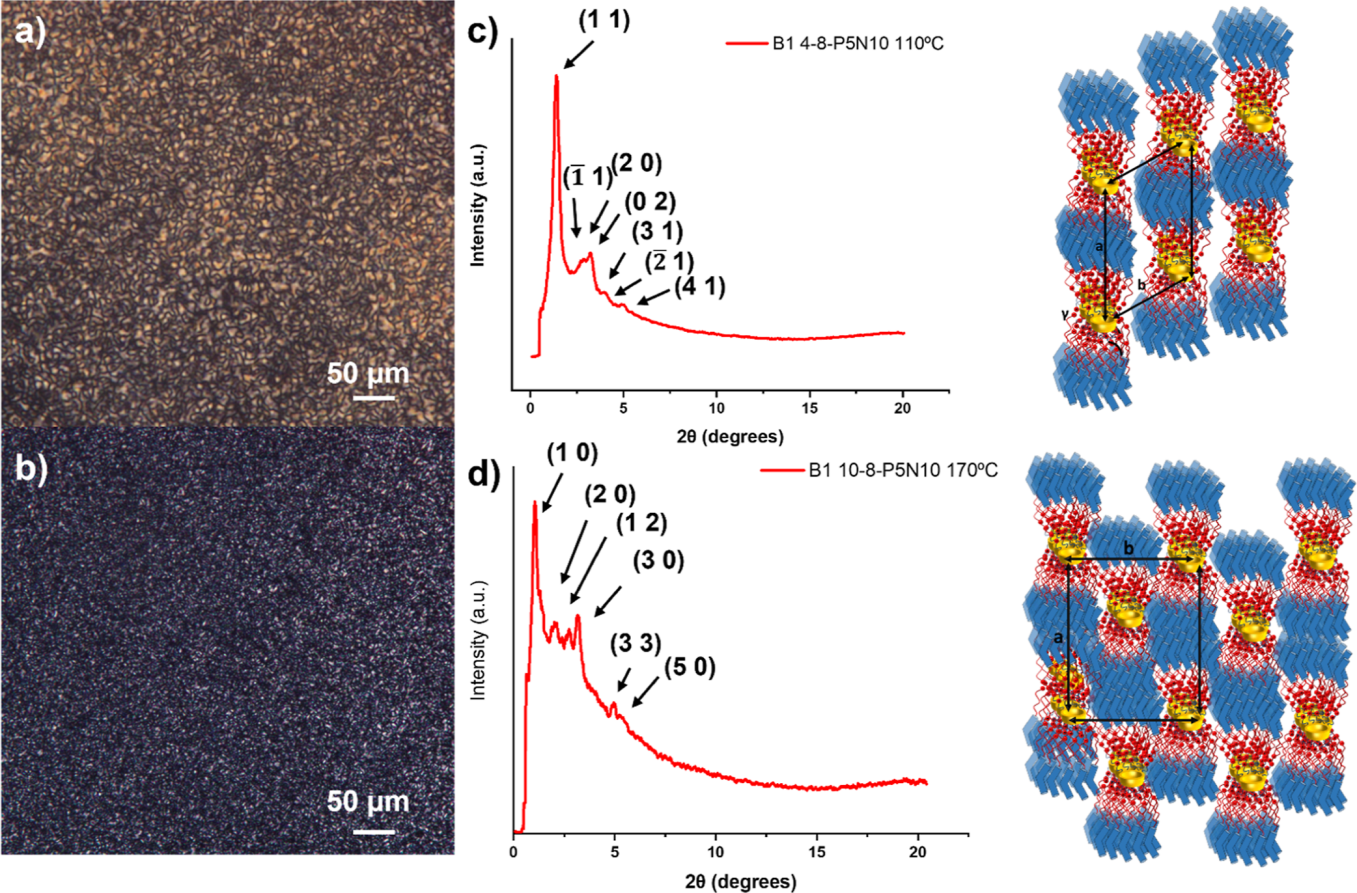
(a) POM microphotograph for **B1 4-8-P5N10** at 85 °C. (c) XRD diffractogram (intensity represented in linear scale) and schematic representation of the molecular organizations for the Col_ob_ mesophase. (b) POM microphotograph for **B1 10-8-P5N10 at** 130 °C. (d) XRD diffractogram (intensity represented in linear scale) and schematic representation of the molecular organizations for the Col_r_ mesophase.

As can be seen in Table 1, the TGA studies revealed that in all cases, there were decomposition temperatures higher than the transition temperature to isotropic liquid. The study of the mesomorphic character of the ionic complexes was carried out by POM observations and DSC thermograms. POM studies of all ionic materials showed fluidity and birefringent textures in a wide range of temperatures before clearing to isotropic liquid. As can be seen in Figure 4 and the Supporting Information (Figure S15a–f), granular textures have been observed in the six ionic complexes which are compatible with the mesophase formation.^42,43,50,51^ It is important to remark than none of the precursor BC acids show liquid crystal behavior.^31,33^

**Table 1:** Phase Transitions and Decomposition Temperatures of Ionic BC Pillar[5]arenes

compound	*T*_2_%^a^ (°C)	phase transitions [°C (Δ*H*, kJ/mol)]^b^
**B1 4-8-P5N10**	232	Cr 78 (20.9) Col_ob_ 140 I*
**B1 10-8-P5N10**	222	Cr_1_ 118 Cr_2_ 126 (8.5) Col_r_ 175 I*
**Bi 4-8-P5N10**	251	Cr 139 (43.5) Col_r_ 170 I*
**Bi 10-8-P5N10**	190	Cr 104 (184.8) Col_r_ 170 I*
**Bazo 4-8-P5N10**	236	Cr 110^c^(17.1) M 220 I*
**Bazo 10-8-P5N10**	194	Cr 120 (117.8) Col_r_ 150 (21.6) I

a Temperature corresponding to a 2% weight loss by TGA.

b DSC data obtained at a rate of 10 °C/min from the second-heating cycle of thermally treated samples. Cr: crystal, Col_r_: rectangular columnar mesophase, Col_ob_: oblique columnar mesophase, M: unidentified mesophase, and I: isotropic liquid. *Data obtained from POM observations.

c Very broad transition.

DSC thermograms (Figures S17 and S18) were carried out to determine thermal and thermodynamic properties of the phase transitions in the ionic complexes (Table 1). In some of the ionic materials, the clearing point was not observed by DSC, and it was determined by POM observations. Furthermore, both POM and DSC studies show in some cases broad transition changes. In previous reports on ionic complexes of this type, materials with –NH_3_^+^/^–^OOC– bonds usually suffered a degradation process after reaching the clearing point, which difficult their workability and prevents them to be used in some applications.^52,53^ However, none of the BC pillar[5]arenes studied here exhibited signs of degradation by POM or DSC experiments after several thermal cycles.

XRD experiments were carried out to determine the type of mesophase formed by the different ionic materials. The temperature at which each experiment was carried out and the resulting data are shown in Table 2. In general, the most conclusive results were obtained on the cooling processes of samples from the clearing point (Figures S19–S25). Lindemann capillaries were used in which materials were introduced by capillarity in the high-temperature liquid phase and cooled immediately to room temperature. Therefore, XRD patterns obtained at room temperature do not necessarily correspond to the ones for a virgin sample.

**Table 2:** XRD Studies of BC Pillar[5]arenes

compound	mesophase^a^	*d* (Å)	Miller index (*hkl*)	structural parameters
**B1 4-8-P5N10**	Col_ob_ (110 °C)	72.82	110	*a* = 74.8 Å
		37.4	1®10	*b* = 66.3 Å
		24.54	200	γ = 55.3°
		24.31	020	
		21.98	310	
		18.43	2®10	
		17.68	410	
**B1 10-8-P5N10**	Col_r_ (170 °C)	83.30	100	*a* = 83.3 Å
		41.65	200	*b* = 69.2 Å
		31.95	120	
		27.76	300	
		17.74	330	
		16.66	500	
**Bi 4-8-P5N10**	Col_r_ (90 °C)	74.60	010	*a* = 78.3 Å
		39.15	200	*b* = 74.6 Å
		26.1	300	
		19.57	400	
		15.66	500	
**Bi 10-8-P5N10**	Col_r_ (70 °C)	46.71	010	*a* = 110.1 Å
		36.70	300	*b* = 46.7 Å
		18.41	600	
		11.47	040	
**Bazo 4-8-P5N10**	M (200 °C)	55.60		
		27.80		
**Bazo 10-8-P5N10**	Col_r_ (140 °C)	90.00	010	*a* = 91.6 Å
		45.00	020	*b* = 90.0 Å
		32.09	220	
		22.19	500	
		13.08	700	

a Thermally treated samples studied in the LCs on the cooling processes.

For the B1-based complexes (Y: –COO−), different mesophases are formed, depending on the length of the flexible spacer. Thus, **B1 4-8-P5N10**, with a short spacer, displayed reflections at low angles that were indexed in an oblique columnar mesophase at 110 °C (Figure 4c). The observed spacing data allowed to obtain the cell parameters *a* and *b* (74.8 and 66.3 Å, respectively), in good agreement of *a* constant with the extended molecular length estimated by molecular models (83.4 Å), with an angle γ of 55.3° at 110 °C (Table 2). The XRD diagram remains essentially the same in the whole temperature range with some variation of the lattice parameters. Figure S21 shows the XRD diagram obtained at room temperature. The broad reflection obtained at high angles corresponds to the diffuse scattering characteristic of the mesophases caused by the alkyl chain interactions. In contrast, **B1 10-8-P5N10**, with a long spacer, displayed typical reflections of a crystalline material at room temperature and showed at 170 °C six reflections at low angles that correspond to a rectangular columnar mesophase with lattice parameters *a* = 83.3 Å and *b* = 69.2 Å (Table 2 and Figure 4d).

Pillar[5]arene complexes based on the lateral Bi structure exhibited in the liquid crystal phase multiple reflections in the low-angle region that can be indexed in a rectangular columnar mesophase (Figures S19, S22, and S23). XRD diffractograms of raw samples of **Bi 4-8-P5N10** (at 25 °C) showed a crystalline nature during heating until 140 °C, while cooling from the isotropic liquid (110, 90, and 60 °C) exhibited mesophase character with multiple reflections that indexed in a rectangular columnar structure with parameter *a* = 78.3 Å (Figure S22). On the other hand, **Bi 10-8-P5N10** exhibited crystalline reflections at room temperature in the raw sample. However, in the cooling process after reaching the clearing point (at 90, 70, and 50 °C), the material also showed mesophase character and retained the rectangular columnar organization in the whole cooling process, with *a* = 110.1 Å (Figure S23).

A longer *a* parameter for **Bi 10-8-P5N10** [110.1 Å (theoretically 117 Å in the most extended conformation)] is expected according to a longer spacer; the *a* parameter is 78.3 Å for **Bi 4-8-P5N10** (theoretically 85 Å). However, the *b* distance for the long spacer compound was shorter (46.7 Å) compared to the *b* constant for the short spacer compound (74.6 Å) (Table 2). These values suggest a different molecular arrangement in the mesophase, despite the same symmetry. It can be concluded that the molecules of **Bi 10-8-P5N10** extend mainly along the *a* axis of the rectangular lattice, whereas the molecules of **Bi 4-8-P5N10** spread out along the two axes.

As in the B1-based complexes, also for Bazo-based ones, different structures have been observed depending on the flexible spacer length. Thus, the XRD diffractogram of **Bazo 4-8-P5N10** is characteristic of a crystalline phase (Figure S24). Only patterns recorded at high temperatures, around 200 °C, were consistent with a mesophase formation, showing one main peak and a weak second-order reflection corresponding to a spacing of 55.6 Å, preventing a definitive mesophase identification (Figure S19c). On the other hand, thermally treated samples of **Bazo 10-8-P5N10** exhibited a pattern characteristic of a rectangular columnar mesophase over a wide range of temperatures, from 120 °C to the isotropic phase on heating and in the whole range of temperatures during cooling (Figures S19d, S20, and S25). The multiple reflections observed at low angles were indexed to a rectangular lattice with the following cell parameters: *a* = 91.6 Å and *b* = 90.0 Å.

This approach to develop ionic BC liquid crystals based on a pillar[5]arene functionalized with terminal amino groups in its ten lateral chains has made it possible to accommodate ten BC derivatives to a cylindrical-shaped pillar[5]arene scaffold in one step. All synthesized complexes have presented mesogenic properties, generally of a columnar type, in contrast to what was observed in other liquid crystal pillar[*n*]arenes, previously described, that showed nematic or lamellar mesophase.^13,20,21,23^

If we compare the liquid crystal properties of these ionic pillar[5]arene complexes with other ionic complexes based on similar BC carboxylic acid units, but with PPI 4-branched and 8-branched dendritic structures, some interesting differences can be pointed. Thus, whereas the ionic dendritic derivatives showed a tendency to crystallize in the cooling process,^31^ the ionic pillar[5]arene derivatives kept the mesophase structure showing a vitrification process in most of the materials. Besides, the ionic PPI derivatives showed SmCP and HNF mesophases instead of columnar ones observed in the ionic pillar[5]arene complexes.^31,32,44,54^

### 2.3 Proton Conduction Properties

The combination of the ionic nature, mesomorphic properties, and thermal stability in the novel BC pillar[5]arenes turns them into highly attractive materials for ion conduction applications.^55−58^ To assess the proton conductivity of these materials, electrochemical impedance spectroscopy (EIS) was employed. The samples were melted up to isotropic liquid between two ITO-coated electrodes and subsequently slowly cooled to room temperature (0.05 °C/min), favoring uniform homeotropic alignment across extensive areas produced by the surface treatment of the ITO. This alignment process holds particular significance in anisotropic materials like LCs, since a better alignment enhances proton conductivity by a higher macroscopic degree of order in mesophases as Col_ob_ and Col_r_ (see Supporting Information Section 1 for further details).

All of the ionic materials were measured from room temperature to the isotropic liquid point and down (heating and cooling runs). The alignment of the materials was tested by POM to verify an appropriate alignment in the cell (see Figure S16) showing important differences with the studies in nonaligned cells previously described. Thus, while the observations in these last cells revealed granular textures associated with columnar mesophases, the textures observed between ITO-treated glasses were less birefringent and undefined, indicating an important surface effect on the order of the materials. This phenomenon has been described before for other BC materials, manifesting the influence of rubbed surfaces in the planar alignment.^59,60^

**Figure 5:**
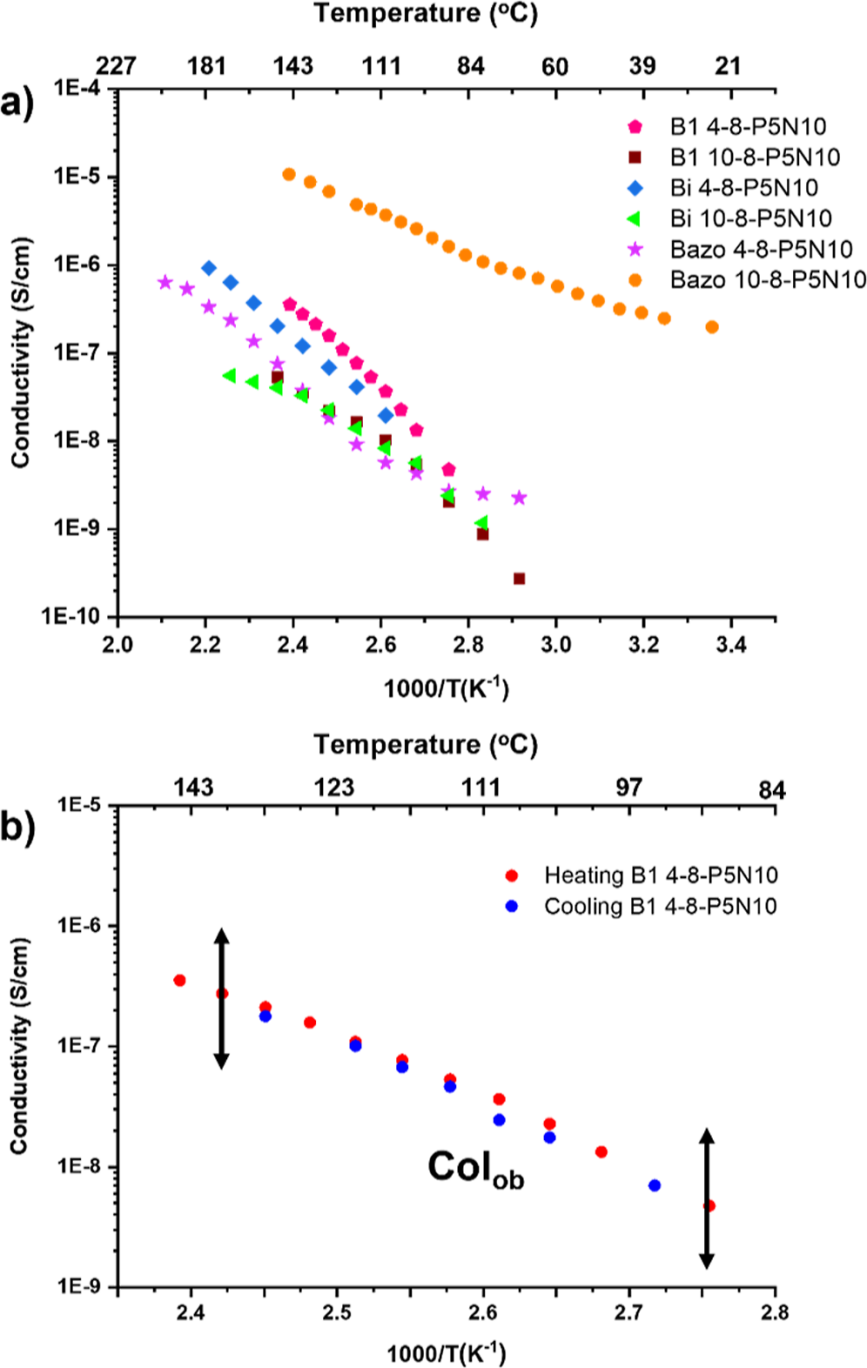
a) Proton conduction variation with temperature in all ionic compounds (arrows enclose the liquid crystal range). (b) Representative proton conduction variation for a heating–cooling process in **B1 4-8-P5N10**.

Figure 5a shows the proton conductivity variation with the temperature for all ionic compounds. We attribute this conduction to proton conduction due to the highly resistive nature of the charge transfer at the electrodes (sample—ITO), as indicated by a pronounced spike at low frequency in the Nyquist plots (Figure S26), suggesting ionic conduction within the samples. All samples showed conductivity that increases with temperature, with decreasing slope as temperature increases for conductivity versus inverse temperature semilogarithmic plots. As temperature increases, the mobility of the liquid crystal phases increases and/or the proton jump between sites is thermally activated.

Compound **Bazo 10-8-P5N10** exhibited the highest proton conduction in all range of temperatures from 25 °C to isotropic temperature (ranging from 10^–7^ to 10^–5^ S/cm in the heating run) with apparent activation energy (Ea) around 0.5–0.6 eV. These values indicate that the dimensions of the unit cell of the compound, its alignment in the cell, and their ionic pair disposition are the most suitable for better proton mobility. During the cooling phase (Figure S27f), the conductivity decreased at temperatures below 100 °C, showing a higher activation energy [the estimated Ea at 50 °C (from the tangent) is 1.3 eV]. Moreover, XRD patterns (Figures S20–S25) revealed a material that retained the Col_r_ arrangement in the whole range of temperatures on cooling from the liquid state until room temperature.

For its homologue with a shorter spacer, **Bazo 4-8-P5N10**, XRD experiments revealed a crystalline structure until high temperature (167 °C). The presence of a nonmesomorphic structure might be reflected in the observed lower proton mobility values. In the cooling run, the conductivity diverges from the heating curve at temperatures below 120 °C, exhibiting lower conductivity. This corresponds to an activation energy (Ea) of 1.33 ± 0.03 eV between 95 and 140 °C (in the cooling run), which is comparable to that of **Bazo 10-8-P5N10**.

In the case of biphenyl derivatives, **Bi 4-8-P5N10** and **Bi 10-8-P5N10**, the compound with a shorter spacer exhibited higher conductivity values (1 order of magnitude higher, around 1.5 × 10^–7^ S/cm, at 140 °C and up to 10^–6^ S/cm at 180 °C) than its larger counterpart with the longer spacer. Their activation energies (Ea’s) in the low-temperature region are around 0.80 eV (Figure S27).

This disparity in the conductivity value might indicate that the shorter “*a*” parameter of **Bi 4-8-P5N10** brings the ionic pairs closer, promoting an easier transfer of protons and enhancing the conductivity values. A more compact arrangement for the smaller compound (shorter spacer) would then make the proton jump between molecules along the conduction direction easier as well as enhance the density of available carriers. For **B1 4-8-P5N10** and **B1 10-8-P5N10**, the behavior is the same as for the biphenyl compounds: the shorter spacer leads to better conductivity values (1 order of magnitude higher, around 3 × 10^–7^ S/cm, at 140 °C), with activation energies at low temperature around 1.00–1.10 eV (Figure S27). Notably, these compounds exhibit different mesophases: Col_ob_ for **B1 4-8-P5N10** and Col_r_ for **B1 10-8-P5N10**. This divergence in the mesophase suggests that the Col_ob_ mesophase offers dense packing within the unit cell, accommodating a higher number of ionic pairs and, consequently, leading to enhanced conductivity values. This difference in the conductivity was also favored by smaller cell parameters for **B1-4-8-P5N10** gathering the ionic pairs and favoring proton mobility.

It is important to note that these proton conductivity values are similar to the values previously described in pillar[5]arene-based ionic complexes but functionalized with simple carboxylic acids in one of their terminal positions, so in this type of molecular designs, no neat differences seem to come from the type of mesophases.^23^

The usual impedance response, Nyquist plots, was obtained in the range of temperatures studied with similar conductivity values to other LCs described previously^61−63^ (see Figure S26). Interestingly, the reproducibility of the measurements and thermal stability during the heating–cooling-measurement process for these materials are worth a comment. Some of the ionic compounds with the carboxylate–ammonium units are known to suffer degradation at high temperatures or transformation of the ionic pairs into amide groups.^64^ This loss of ionic bonds results in a lower conductivity during the cooling process. For this reason, all materials were studied during the heating–cooling processes. Figure 5b, as an example, shows the complete cycle for **B1 4-8-P5N10**. It is to note that this material, **Bi 4-8-P5N10**, and the **Bazo** compounds down to 100 or 120 °C exhibited the same conductivity values for each temperature in both up and down runs (see Figure S27). This fact allows us to affirm that they did not suffer any degradation after reaching the liquid transition temperature. However, in some of the BC pillar[5]arenes studied, the conductivity values obtained in the cooling run are slightly lower than in the heating measurements over a large temperature range (see Supporting Information Section 3.6 and Figure S27). The XRD diffractograms showed that the ordering (mesophase or crystalline) is different in the heating or cooling runs (Section 2.2 and Figures S19–25), and thus, differences in absolute values of conductivity are not unexpected. The much higher conductivity in the heating run at low temperatures together with the much lower apparent activation energy of **Bazo 4-8-P5N10** and perhaps some contribution to **Bazo 10-8-P5N10** could have also participation of extra charge carriers (H^+^ or OH^–^) caused by humidity in the sample.^65,66^

It is well known that the presence of photoresponsive azobenzene units can be used to modulate conductive properties by *trans*–*cis* isomerization processes.^61,67−69^ The exposition of the cells of **Bazo 10-8-P5N10** and **Bazo 4-8-P5N10** materials to UV light of 325 nm produced the photoisomerization of the azobenzene unit of the BC moieties, as observed by UV spectroscopy with a significant decrease in the intensity of the *n*–π* band and an increase in the intensity of the π–π* band at 500 nm (see the Supporting Information for further details, Figure S28). The isomerization reverts to the original state upon exposure to light or after some hours. Before coming back to the *trans* state, the proton conductivity of the compounds was measured again (after 0 h and after 24 h since irradiation). Interestingly, in the irradiated cells, the proton mobility disappeared even after 24 h irradiation. The isomerization of the azobenzene structure seems to produce a disturbance^70,71^ that modifies the column packing, eliminating the long-distance order that benefits the proton mobility.

### 2.4 Self-Assembly in Aqueous Solution

The preparation of aggregates in aqueous solution has been widely described for a large number of polymers, ionic molecules, and host guest complexes, leading to the formation of different kinds of morphologies (nanotubes, fibers, micelles...).^72−75^ In this context, a crucial factor is the amphiphilic character of the materials. These ionic BC pillar[5]arenes contain ten ionic pairs as a hydrophilic part, which combine with the hydrophobic character of the pillar[5]arene and BC structure. The aggregates were prepared by the cosolvent method, dissolving the ionic pillar[5]arenes in THF and slowly adding water. The turbidity of the solution was followed by UV (see Supporting Information Figure S29 for further information), then the sample was dialyzed against water to remove THF using a cellulose membrane with a molecular weight cutoff of 1000 kDa.

The aggregates were studied by TEM, and the images clearly showed the formation of nanostructures for all ionic derivatives, providing different morphologies. These data are collected in Table 3.

**Table 3:** Morphology and Dimension Details of the Ionic BC Pillar[5]arene Aggregates in Water Solution after Dialysis

compound	morphology of the aggregates	dimensions of the aggregates^a^
**B1 4-8-P5N10**	nanotubes	Φ_outer_: 250 nm
		Φ_inner_: 180 nm
**B1 10-8-P5N10**	nanotubes	Φ_outer_: 225 nm
		Φ_inner_: 160 nm
**Bi 4-8-P5N10**	helical nanofilaments	*w*: 60–67 nm
		*p*: 70–80 nm
		*d*: 5–6 nm
**Bi 10-8-P5N10**	intertwined twisted fibers	*w*: 32–34 nm
		*p*: 58–74 nm
**Bazo 4-8-P5N10**	helical nanofilaments	*w*: 30–40 nm
		*p*: 80–90 nm
		*d*: 5–6 nm
**Bazo 10-8-P5N10**	twisted fibers	*w*: 25–30 nm
		*p*: 70–80 nm

a Φouter: external diameter of nanotubes, Φinner: internal diameter of nanotubes, *w*: width of fibers or ribbons, *p*: half pitch, *d*: layer spacing; estimated by TEM.

Samples were studied before and after dialyzing (Supporting Information Figures S30–S32) to corroborate the influence of water and the evolution of the nanostructures. All ionic materials self-assembled in aqueous media setting up stable aggregates with defined morphology.

**Figure 6:**
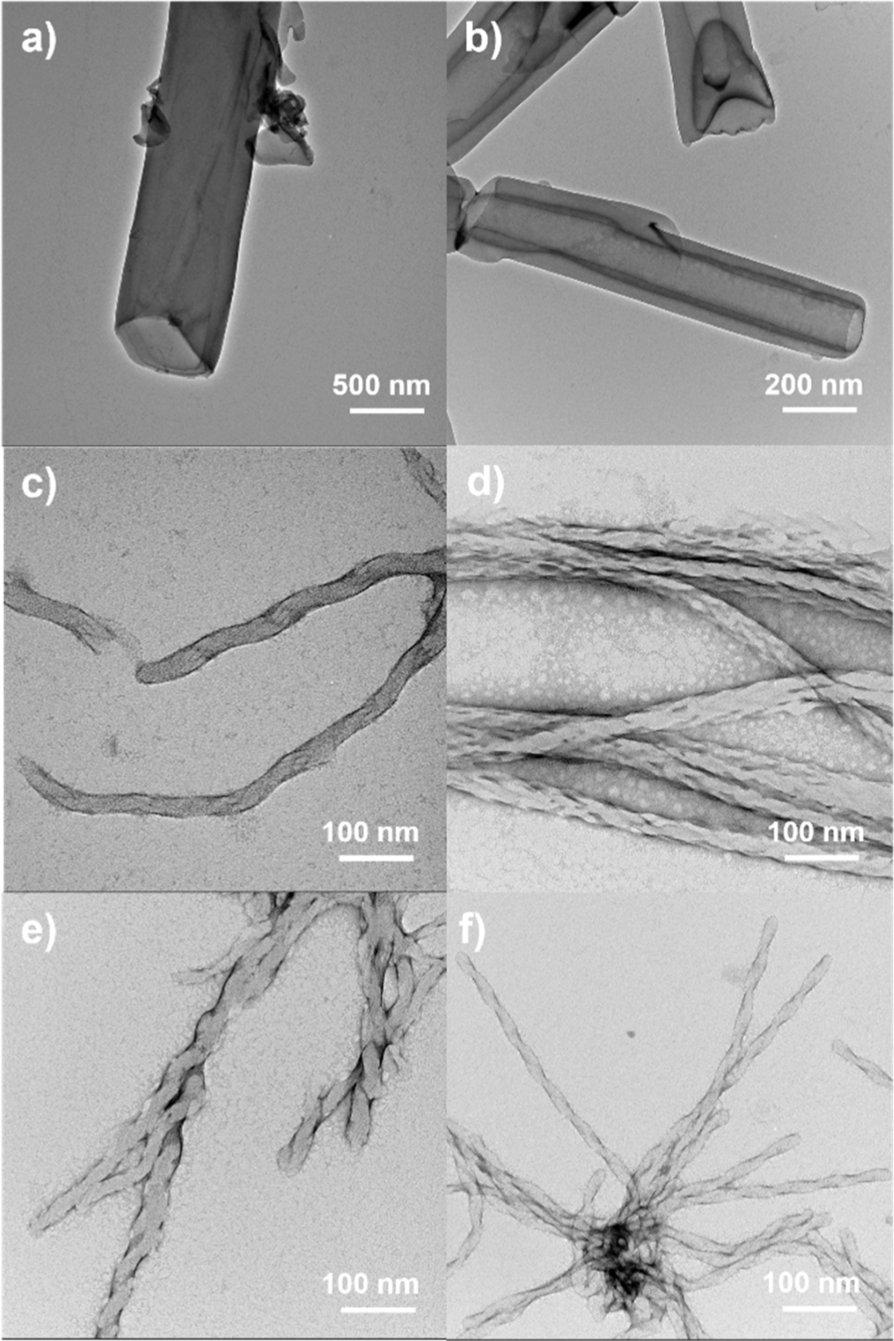
TEM images of dialyzed samples: (a) **B1 4-8-P5N10**, (b) **B1 10-8-P5N10**, (c) **Bi 4-8-P5N10**, (d) **Bi 10-8-P5N10**, (e) **Bazo 4-8-P5N10**, and (f) **Bazo 10-8-P5N10**.

The ionic materials based on the **B1** derivatives were uniquely able to set tubular structures with external diameters around 225–250 nm (Figure 6a,b) and internal ones of 160–180 nm. On the other hand, complexes based on **Bi** and **Bazo** units stabilized chiral fibrillar morphologies; twisted fibers before and helical filaments after dialysis (Figures 6c–f and S31, S32) even with the achiral nature of their molecular designs. In the case of pillar[5]arenes functionalized with BCs containing short spacers, **Bazo 4-8-P5N10** and **Bi 4-8-P5N10**, TEM images showed before and after dialyzing (Figure 6c–e and S31, S32) the presence of dense helical filaments. The supramolecular interaction between molecules, resulting in their stacking in several layers and subsequent water repellence, is noteworthy. In good agreement with other reports, the stacking phenomenon in BC molecules induces chiral twisting in the aggregate without the presence of any chiral center. This is attributed to the accumulation of multiple layers with the BC units tiled with respect to the normal layers.^31,32,45,47^

**Figure 7:**
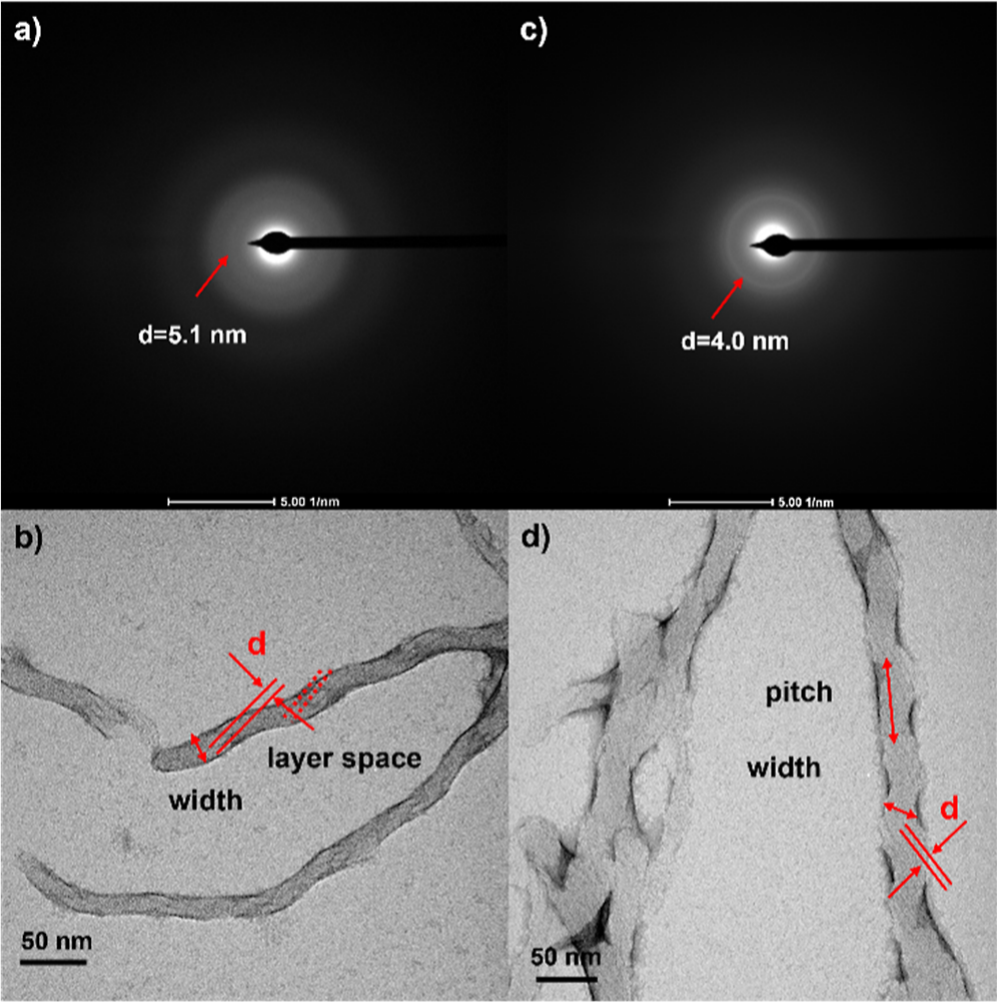
SAED patterns of (a) **Bi 4-8-P5N10** and (c) **Bazo 4-8-P5N10**, and images obtained from TEM, (b) **Bi 4-8-P510** and (d) **Bazo 4-8-P5N10**.

**Figure 8:**
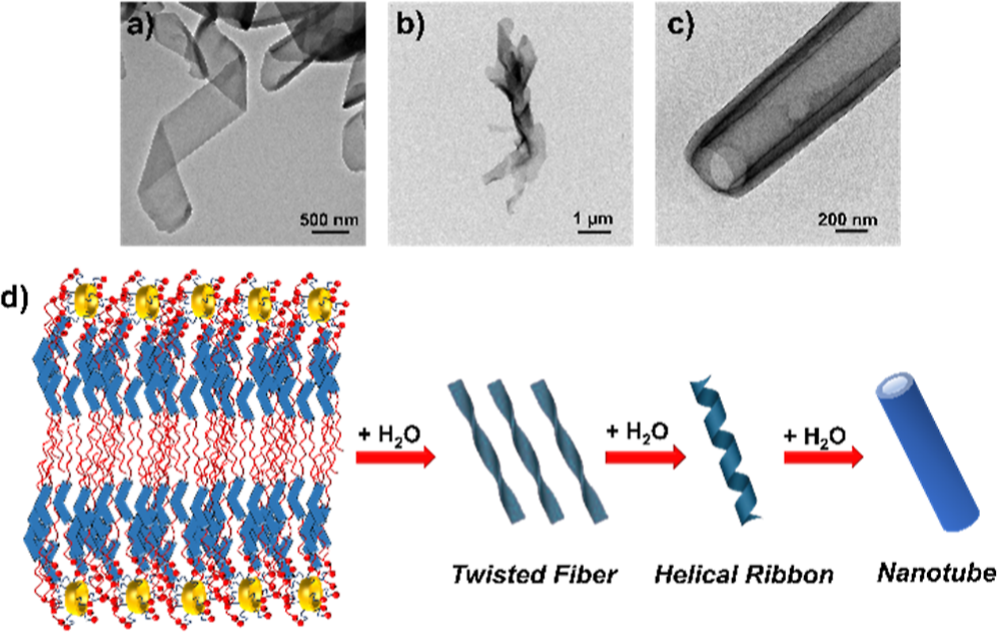
Self-assembly mechanism of nanotubes. TEM images of the process: (a,b) twisted fibers, (c) nanotube, and (d) schematic representation of the process from the molecular assembly to the nanotubes.

To take a close look to the internal structure of the fibers generated by **Bi 4-8-P5N10** and **Bazo 4-8-P5N10**, SAED was carried out (Figure 7a–c), obtaining a spacing distance of around 4–5 nm which are in fair agreement with the distances reported for these kinds of BC-based supramolecular organizations, and the layer estimations from TEM (Figure 7b–d) suggesting a molecular distribution in layers.^32,76,77^ Based on these experiments and the previous results published by our group related with ionic BC materials,^31,32^ we propose a similar molecular organization mechanism: ionic molecules are folded around the pillar[5]arene framework, exposing the ionic pairs to water. Considering that the molecular size of carboxylic acid **Bazo 4-8** is around 2.4 nm and the calculated distance for a bilayer should be 4.8 nm, close to the 4.0 nm from SAED, some interdigitation of the alkyl chains could be proposed. This sequence evolved to multiple layers with important intermolecular interactions between the BC moieties; the resulting structure dominated by the high tendency of the BC units to form tilted scaffolds grows in a twisted way emerging helical structures similar to those described for the singular HNFs (Figure 8d).^31,32^ However, tubular structures could also be proposed for these materials since it cannot be ruled out that the layered structure constructed, instead of twisting along the longitudinal axis of the filament, is wound in a helical manner around the longitudinal axis.

In **Bi 10-8-P5N10**, an intertwining of the fibers was observed, thus reducing their contact with water. The elongated spacer leads to a twist in the aggregate instead of a saddle of molecules with a width of around 30 nm and a pitch of 70–80 nm for the **Bazo** derivative and 58–74 nm for the Bi derivative.

For further knowledge of the evolution in the assembly process in tubular morphologies, different samples of **B1 10-8-P5N10** and **B1 4-8-P5N10** at lower percentage of water were prepared hoping to observe their development from fiber to nanotube. These samples were analyzed by TEM and Figure 8 shows the evolution of the aggregates from fibers (a) to helical ribbon formed by several fibers (b). This ribbon finally evolved to the nanotubes described previously in Figure 6a,b. For better understanding of the self-assembly process, several ^1^H NMR studies were performed: compound **B1 4-8-P5N10** was dissolved in THF-*d*_8_ and the cosolvent method was carried out with deuterated water. After each addition of D_2_O, an NMR spectrum was performed (Figure S13). These studies confirm the strong influence of the solvent in the self-assembly process as the aromatic rings stack closer when the water percentage increased, observing shifts upfield in the aromatic ring’s peaks.

## 3 Conclusions

The ionic interactions between amino-ended pillar[5]arene and BC nonmesogenic carboxylic acids have allowed to obtain six new supramolecular complexes exhibiting new columnar (Col_r_ and Col_ob_) liquid crystal behavior.

In bulk, either solid or mesophase, these materials showed proton conductivity with values similar to those previously described in comparative ionic complexes formed with promesogenic calamitic moieties. Interestingly, the introduction of azobenzene photoisomerizable compounds resulted in a conductivity photoresponse, switching off efficiently the proton mobility in the materials.

In addition, as a result of the ten hydrophilic ionic pairs created in these ionic complexes and the hydrophobic character of the BC units and the central pillar[5]arene moiety, these materials possess a noticeable amphiphilic character. Their aggregation in water has provided an extensive analysis about the relationship between the BC structure, the spacer length to the ionic moiety, and the morphology of the nanostructured aggregates. As a result, twisted fibers are obtained for azobenzene and biphenyl units connected through long spacers and chiral helical nanofilaments for short spacers close to HNF-type mesophases of BC compounds. In the case of ester-based BCs, nanotubes were obtained for both spacers. This analysis has supplied information about the structural details and the assembly process, demonstrating the suitability of pillar[5]arenes to accommodate BC units leading to a large variety of supramolecular functional nanostructures. Depending on the environment, bulk, or polar solvent, these ionic molecules adopt lamellar arrangements but rather different molecular dispositions according to their amphiphilic nature and BC packing driving forces. These results open new and very attractive possibilities for the design of nanostructured multifunctional materials, where through the chemistry of materials, the functional characteristics of innovative advanced materials can be synergistically combined by the appropriate design of the BC units and the host capacity of the pillar[5]arene unit.

## 4 Experimental and Method Section

### 4.1 Materials

#### 4.1.1 General Procedure for the Preparation of Ionic Complexes

The synthesis of different acids and pillar[5]arene **P5N10** was carried out following the antecedent papers. Ionic dendrimers were prepared by following the previously described methodology. A solution of the corresponding acid in dry tetrahydrofuran was added to a solution of the pillar[5]arene **P5N10**, in approximately 1:1 (primary amine groups/carboxylic acid groups) stoichiometry. The mixture was ultrasonicated for 60 min, and then it was slowly evaporated at room temperature and dried in vacuum at 40 °C until the weight remained constant. Experimental details are available in the Supporting Information.

#### 4.1.2 General Procedure for the Preparation of Aggregates

For the preparation of the self-assemblies, a solution of 5 mg mL^–1^ of the amphiphilic ionic BC pillar[5]arene in THF was prepared, and Milli-Q water was gradually added, while self-assembly was followed by measuring the turbidity in UV. When a critical water content was reached, a high increase in turbidity happened, indicating that the self-assembling process took place. Once turbidity reached an almost constant value, the mixture was dialyzed against water to remove the organic solvent by using a Spectra/Por dialysis membrane (MWCO 1000) for 3 days. Water suspensions of the aggregates with a concentration of around 2 mg mL^–1^ were obtained.

#### 4.1.3 Irradiation Experiments

Cells containing materials **Bazo 10-8-P5N10** and **Bazo 4-8-P5N10** were irradiated with an LED of 325 nm with an intensity of 300 mW/cm^2^ at room temperature for 5 min.

### 4.2 Characterization Techniques

#### 4.2.1 Chemical Characterization

^1^H NMR and ^13^C NMR spectra were acquired on a Bruker AV400 spectrometer. The experiments were performed at room temperature in different deuterated solvents (CDCl_3_, CD_2_Cl_2_, or DMSO-*d*_6_). Chemical shifts are given in ppm relative to TMS and the solvent residual peak was used as the internal standard. Infrared spectra were recorded on a Bruker VERTEX 70 FT-IR spectrometer. The samples were prepared on KBr pellets with a concentration of the product of 1–2% (w/w). Mass spectra were obtained on a MICROFLEX Bruker (MALDI^+^) spectrometer with a dithranol matrix. UV–vis absorption spectra were recorded on an ATI-Unicam UV4–200 spectrophotometer.

*Mesogenic behavior* was investigated by POM using an Olympus BH-2 polarizing microscope fitted with a Linkam THMS600 hot stage. TGA was performed using a Q5000IR from TA Instruments at a heating rate of 10 °C min^–1^ under a nitrogen atmosphere. Thermal transitions were determined by DSC using a DSC Q2000 from TA Instruments with powdered samples (2–5 mg) sealed in aluminum pans. Glass transition temperatures (*T*_g_) were determined at the half height of the baseline jump, and first-order transition temperatures were read at the maximum of the corresponding peak. XRD diagrams were recorded using a STOE STADIVARI goniometer equipped with a Genix3D microfocus generator (Xenocs) and a Dectris Pilatus 100 K detector. Temperature control was achieved using a nitrogen-gas Cryostream controller (Oxford Cryosystems) allowing for a temperature control of about 0.1 °C. Lindemann capillaries of diameter 0.6 mm were utilized. Monochromatic Cu Kα radiation (λ = 1.5418 Å) was used. The exposure time was 2 min.

*Microscopy analysis (TEM)* was performed using an FEI Tecnai T20 microscope (FEI Company, Waltham, MA, USA) operating at 200 kV. TEM samples were prepared by adding 10 μL of each self-assembly dispersion at an approximately 1.0 mg mL^–1^ concentration on a continuous carbon film-copper grid, and the excess was removed by capillarity using filter paper. Then, the grids were stained with uranyl acetate (1% aqueous solution), removing the excess again by capillarity using filter paper.

*EIS* was recorded with a frequency response analyzer, Solartron SI1260A, from AMETEK in the frequency range from 1 Hz to 1 MHz (amplitude of the applied voltage: 50 mV). The temperature of the sample was controlled with a Linkam THMS600 hot stage. The conductivities were studied as a function of temperature between 30 °C and isotrope temperature at different intervals in the heating and in the cooling runs. At each step, the temperature was held until equilibration before measurement. On average, the net heating and cooling rates were between 1 and 3 °C/min. For the preparation of the cells for ionic conductivities, the appropriate amount of the ionic compound was placed into an ITO electrode that was sandwiched with another ITO electrode by controlling the thickness by using glass spacers (10–20 μm). The cell was heated up to a few degrees above the melting point of the liquid crystal, and the cell was pressed to obtain the thin film. The measured impedance spectra were plotted in complex plane plots, imaginary (*Z*″) versus real (*Z*′) component. They consist of a high frequency arc due to the sample electrical response, more or less overlapped with the low frequency electrode contribution. This assignment was made according to their equivalent capacitances. The sample resistance (*R*_b_) was estimated from the minimum of the −*Z*″ vs *Z*′ at the low frequency side of the high frequency arc (sample contribution). The conductivities, assigned to proton conductivities, σ (S·cm^–1^), were calculated with the formula: σ = [*d*/(*R*_b_·*A*)], where *d* (cm) is the thickness of the film, *A* (cm^2^) is the area of the film, and *R*_b_ (Ω) is the resistance of the sample. After the preparation of the cell, a random orientation of the mesophase was observed between the electrodes. Samples were mechanically sheared within the cell (in order to obtain an alignment of the molecules) at isotropic temperature and then slowly cooled down to room temperature (0.05 °C min^–1^).

## Supporting Information Available

Materials and characterization techniques, experimental procedures, NMR spectra, FTIR spectra, POM textures, DSC thermograms, X-ray diffractograms, EIS measurements, UV–vis spectra, and TEM images (PDF)

## References

[ref1] Ogoshi T.; Kanai S.; Fujinami S.; Yamagishi T.-A.; Nakamoto Y. para-Bridged Symmetrical Pillar[5]arenes: Their Lewis Acid Catalyzed Synthesis and Host-Guest Property. J. Am. Chem. Soc. 2008, 130, 5022–5023. 10.1021/ja711260m. 18357989

[ref2] Strutt N. L.; Forgan R. S.; Spruell J. M.; Botros Y. Y.; Stoddart J. F. Monofunctionalized Pillar[5]arene as a Host for Alkanediamines. J. Am. Chem. Soc. 2011, 133, 5668–5671. 10.1021/ja111418j. 21443238

[ref3] Kakuta T.; Yamagishi T.-A.; Ogoshi T. Stimuli-Responsive Supramolecular Assemblies Constructed from Pillar[n]arenes. Acc. Chem. Res. 2018, 51, 1656–1666. 10.1021/acs.accounts.8b00157. 29889488

[ref4] Ogoshi T.; Masaki K.; Shiga R.; Kitajima K.; Yamagishi T.-A. Planar-Chiral Macrocyclic Host Pillar[5]arene: No Rotation of Units and Isolation of Enantiomers by Introducing Bulky Substituents. Org. Lett. 2011, 13 (5), 1264–1266. 10.1021/ol200062j. 21288006

[ref5] Hu X.-B.; Chen Z.; Chen L.; Zhang L.; Hou J.-L.; Li Z.-T. Pillar[n]arenes (n = 8–10) with two cavities: synthesis, structures and complexing properties. Chem. Commun. 2012, 48, 10999–11001. 10.1039/c2cc36027f. 23038422

[ref6] Ogoshi T.; Yamagishi T.-A. Pillararenes: Versatile Synthetic Receptors for Supramolecular Chemistry. Eur. J. Org Chem. 2013, 2013, 2961–2975. 10.1002/ejoc.201300079.

[ref7] Zyryanov G. V.; Kopchuk D. S.; Kovalev I. S.; Santra S.; Majee A.; Ranu B. C. Pillararenes as Promising Carriers for Drug Delivery. Int. J. Mol. Sci. 2023, 24, 5167 10.3390/ijms24065167. 36982244 PMC10049520

[ref8] Jiang L.; Huang X.; Chen D.; Yan H.; Li X.; Du X. Supramolecular Vesicles Coassembled from Disulfide-Linked Benzimidazolium Amphiphiles and Carboxylate-Substituted Pillar-[6]arenes that Are Responsive to Five Stimuli. Angew. Chem., Int. Ed. 2017, 56, 2655–2659. 10.1002/anie.201611973. 28140489

[ref9] Zhu H.; Li Q.; Zhu W.; Huang F. Pillararenes as Versatile Building Blocks for Fluorescent Materials. Acc. Mater. Res. 2022, 3, 658–668. 10.1021/accountsmr.2c00063.

[ref10] Wang K.; Zhang R.; Song Z.; Zhang K.; Tian X.; Pangannaya S.; Zuo M.; Hu X.-Y. Dimeric Pillar[5]arene as a Novel Fluorescent Host for Controllable Fabrication of Supramolecular Assemblies and Their Photocatalytic Applications. Adv. Sci. 2023, 10, 2206897 10.1002/advs.202206897. PMC10037968 36683255

[ref11] Hua B.; Shao L.; Zhang Z.; Liu J.; Huang F. Cooperative Silver Ion-Pair Recognition by Peralkylated Pillar[5]arenes. J. Am. Chem. Soc. 2019, 141, 15008–15012. 10.1021/jacs.9b08257. 31509696

[ref12] Chen J.-F.; Ding J.-D.; Wei T.-B. Pillararenes: fascinating planar chiral macrocyclic arenes. Chem. Commun. 2021, 57, 9029–9039. 10.1039/D1CC03778A. 34498646

[ref13] Fa S.; Mizobata M.; Nagano S.; Suetsugu K.; Kakuta T.; Yamagishi T.-a.; Ogoshi T. Reversible “On/Off” Chiral Amplification of Pillar[5]arene Assemblies by Dual External Stimuli. ACS Nano 2021, 15, 16794–16801. 10.1021/acsnano.1c06975. 34542992

[ref14] Andrei I.-M.; Strilets D.; Fa S.; Baaden M.; Ogoshi T.; Barboiu M. Combinatorial Screening of Water/Proton Permeation of Self-Assembled Pillar[5]arene Artificial Water Channel Libraries. Angew. Chem., Int. Ed. 2023, 62, e202310812 10.1002/anie.202310812. 37610532

[ref15] Wu Y.; Tang M.; Wang Z.; Shi L.; Xiong Z.; Chen Z.; Sessler J. L.; Huang F. Pillararene incorporated metal-organic frameworks for supramolecular recognition and selective separation. Nat. Commun. 2023, 14, 4927 10.1038/s41467-023-40594-2. 37582786 PMC10427641

[ref16] Donnio B.; Buathong S.; Bury I.; Guillon D. Liquid crystalline dendrimers. Chem. Soc. Rev. 2007, 36, 1495–1513. 10.1039/b605531c. 17660881

[ref17] Sun H. J.; Zhang S.; Percec V. From structure to function via complex supramolecular dendrimer systems. Chem. Soc. Rev. 2015, 44, 3900–3923. 10.1039/C4CS00249K. 25325787

[ref18] Kato T.; Kihara H.; Kumar U.; Uryu T.; Fréchet J. M. J. A Liquid-Crystalline Polymer Network Built by Molecular Self-Assembly through Intermolecular Hydrogen Bonding. Angew. Chem., Int. Ed. 1994, 33, 1644–1645. 10.1002/anie.199416441.

[ref19] Kato T.; Ihata O.; Ujiie S.; Tokita M.; Watanabe J. Self-Assembly of Liquid-Crystalline Polyamide Complexes through the Formation of Double Hydrogen Bonds between a 2,6-Bis(amino)pyridine Moiety and Benzoic Acids. Macromolecules 1998, 31 (11), 3551–3555. 10.1021/ma9719014.

[ref20] Concellón A.; Romero P.; Marcos M.; Barberá J.; Sánchez-Somolinos C.; Mizobata M.; Ogoshi T.; Serrano J. L.; Del Barrio J. Coumarin-Containing Pillar[5]arenes as Multifunctional Liquid Crystal Macrocycles. J. Org. Chem. 2020, 85, 8944–8951. 10.1021/acs.joc.0c00852. 32545956

[ref21] Nierengarten I.; Guerra S.; Holler M.; Karmazin-Brelot L.; Barberá J.; Deschenaux R.; Nierengarten J.-F. Macrocyclic Effects in the Mesomorphic Properties of Liquid-Crystalline Pillar[5]- and Pillar[6]arenes. Eur. J. Org Chem. 2013, 2013, 3675–3684. 10.1002/ejoc.201300356.

[ref22] Nierengarten I.; Guerra S.; Aziza H. B.; Holler M.; Abidi R.; Barberá J.; Deschenaux R.; Nierengarten J.-F. Piling Up Pillar[5]arenes To Self-Assemble Nanotubes. Chem. - Eur. J. 2016, 22, 6185–6189. 10.1002/chem.201600688. 26888329

[ref23] Marín I.; Merino R. I.; Barberá J.; Concellón A.; Serrano J. L. Ionic self-assembly of pillar[5]arenes: proton-conductive liquid crystals and aqueous nanoobjects with encapsulation properties. Mater. Adv. 2023, 4, 5564–5572. 10.1039/D3MA00698K.

[ref24] Marcos M.; Martín-Rapún R.; Omenat A.; Serrano J. L. Highly congested liquid crystal structures: dendrimers, dendrons, dendronized and hyperbranched polymers. Chem. Soc. Rev. 2007, 36, 1889–1901. 10.1039/b611123h. 17982516

[ref25] Hernández-Ainsa S.; Marcos M.; Serrano J. L. Dendrimeric and hyperbranched liquid crystal structures. In Handbook of Liquid Crystals; Goodby J. W., Collings P. J., Kato T., Tschierske C., Gleeson H., Raynes P., Eds.; Wiley-VCH Verlag GmbH & Co. KGaA, 2014; Vol. 7; p 259.

[ref26] Concellón A.; Iguarbe V. Ionic Self-Assembly of Dendrimers. In Supramolecular Assemblies Based on Electrostatic Interactions; Aboudzadeh M. A., Frontera A., Eds.; Springer International Publishing: Cham, 2022; p 85.

[ref27] Goossens K.; Lava K.; Bielawski C. W.; Binnemans K. Ionic Liquid Crystals: Versatile Materials. Chem. Rev. 2016, 116 (8), 4643–4807. 10.1021/cr400334b. 27088310

[ref28] Ruan Q.; Yao M.; Yuan D.; Dong H.; Liu J.; Yuan X.; Fang W.; Zhao G.; Zhang H. Ionic liquid crystal electrolytes: Fundamental, applications and prospects. Nano Energy 2023, 106, 108087 10.1016/j.nanoen.2022.108087.

[ref29] Salikolimi K.; Sudhakar A. A.; Ishida Y. Functional Ionic Liquid Crystals. Langmuir 2020, 36 (40), 11702–11731. 10.1021/acs.langmuir.0c01935. 32927953

[ref30] Kapernaum N.; Lange A.; Ebert M.; Grunwald M. A.; Haege C.; Marino S.; Zens A.; Taubert A.; Giesselmann F.; Laschat S. Current Topics in Ionic Liquid Crystals. ChemPlusChem 2022, 87, e202100397 10.1002/cplu.202100397. 34931472

[ref31] Castillo-Vallés M.; Cano M.; Bermejo-Sanz A.; Gimeno N.; Ros M. B. Towards supramolecular nanostructured materials: control of the self-assembly of ionic bent-core amphiphiles. J. Mater. Chem. C 2020, 8, 1998–2007. 10.1039/C9TC06002B.

[ref32] Cano M.; Sánchez-Ferrer A.; Serrano J. L.; Gimeno N.; Ros M. B. Supramolecular Architectures from Bent-Core Dendritic Molecules. Angew. Chem., Int. Ed. 2014, 53, 13449–13453. 10.1002/anie.201407705. 25323567

[ref33] Liebsch J.; Strachan R.; Suthaharan S.; Dominguez-Candela I.; Auria-Soro C.; San-Millan A.; Walker R.; Chilukuri B.; Blanca Ros M.; Martinez-Felipe A. Tailoring the dielectric and ferroelectric response of mixtures containing bent-core liquid crystals through light-irradiation and composition. J. Mol. Liq. 2024, 399, 124371 10.1016/j.molliq.2024.124371.

[ref34] Etxebarria J.; Blanca Ros M. Bent-core liquid crystals in the route to functional materials. J. Mater. Chem. 2008, 18, 2919–2926. 10.1039/b803507e.

[ref35] Reddy R. A.; Tschierske C. Bent-Core Liquid Crystals: Polar Order, Superstructural Chirality and Spontaneous Desymmetrisation in Soft Matter Systems. J. Mater. Chem. 2006, 16 (10), 907–961. 10.1039/B504400F.

[ref36] Tschierske C. Development of Structural Complexity by Liquid-Crystal Self-Assembly. Angew. Chem., Int. Ed. 2013, 52 (34), 8828–8878. 10.1002/anie.201300872. 23934786

[ref37] Takezoe H.; Takanishi Y. Bent-Core Liquid Crystals: Their Mysterious and Attractive World. Jpn. J. Appl. Phys. 2006, 45, 597–625. 10.1143/JJAP.45.597.

[ref38] Eremin A.; Jákli A. Polar Bent-Shape Liquid Crystals - From Molecular Bend to Layer Splay and Chirality. Soft Matter 2013, 9 (3), 615–637. 10.1039/C2SM26780B.

[ref39] Imrie C. T.; Luckhurst G. R. In Handbook of Liquid Crystals; Goodby J. W., Collings P. J., Kato T., Tschierske C., Gleeson H., Raynes P., Eds.; Wiley-VCH: Weinheim, 2014 (Chapters 3, 4, 5 and 8 are focused on bent-core liquid crystals).

[ref40] Takezoe H. Polar Liquid Crystals-Ferro, Antiferro, Banana, and Columnar. Mol. Cryst. Liq. Cryst. 2017, 646 (1), 46–65. 10.1080/15421406.2017.1284377.

[ref41] Le K. V.; Takezoe H.; Araoka F. Chiral Superstructure Mesophases of Achiral Bent-Shaped Molecules-Hierarchical Chirality Amplification and Physical Properties. Adv. Mater. 2017, 29, 1602737 10.1002/adma.201602737. 27966798

[ref42] Li L.; Salamończyk M.; Shadpour S.; Zhu C.; Jákli A.; Hegmann T. An unusual type of polymorphism in a liquid crystal. Nat. Commun. 2018, 9, 714 10.1038/s41467-018-03160-9. 29459670 PMC5818537

[ref43] Shadpour S.; Nemati A.; Liu J.; Hegmann T. Directing the Handedness of Helical Nanofilaments Confined in Nanochannels Using Axially Chiral Binaphthyl Dopants. ACS Appl. Mater. Interfaces 2020, 12, 13456–13463. 10.1021/acsami.9b20696. 32092259

[ref44] Vergara J.; Gimeno N.; Cano M.; Barberá J.; Romero P.; Serrano J. L.; Ros M. B. Mesomorphism from Bent-Core Based Ionic Dendritic Macromolecules. Chem. Mater. 2011, 23, 4931–4940. 10.1021/cm201809r.

[ref45] Castillo-Vallés M.; Martínez-Bueno A.; Giménez R.; Sierra T.; Ros M. B. Beyond Liquid Crystals: New Research Trends for Mesogenic Molecules in Liquids. J. Mater. Chem. C 2019, 7 (46), 14454–14470. 10.1039/C9TC04179F.

[ref46] Ros M. B. Supramolecular Versatility of Bent-Shaped Molecules. In Supramolecular Nanotechnology: Advanced Design of Self-Assembled Functional Materials; Azzaroni O., Conda-Sheridan M., Eds.; Wiley-VCH Verlag GmbH & Co. KGaA, 2023 (Chapter 24).

[ref47] Gowda A.; Pathak S. K.; Rohaley G. A. R.; Acharjee G.; Oprandi A.; Williams R.; Prévôt M. E.; Hegmann T. Organic chiral nano- and microfilaments: types, formation, and template applications. Mater. Horiz. 2024, 11, 316–340. 10.1039/D3MH01390A. 37921354

[ref48] Sezgin B.; Liu J.; Gonçalves D. P. N.; Zhu C.; Tilki T.; Prévôt M. E.; Hegmann T. Controlling the Structure and Morphology of Organic Nanofilaments Using External Stimuli. ACS Nanosci. Au 2023, 3, 295–309. 10.1021/acsnanoscienceau.3c00005. 37601923 PMC10436377

[ref49] Sun Y.; Zhang F.; Quan J.; Zhu F.; Hong W.; Ma J.; Pang H.; Sun Y.; Tian D.; Li H. A biomimetic chiral-driven ionic gate constructed by pillar[6]arene-based host-guest systems. Nat. Commun. 2018, 9, 2617 10.1038/s41467-018-05103-w. 29976986 PMC6033921

[ref50] Liu J.; Shadpour S.; Prévôt M. E.; Chirgwin M.; Nemati A.; Hegmann E.; Lemieux R. P.; Hegmann T. Molecular Conformation of Bent-Core Molecules Affected by Chiral Side Chains Dictates Polymorphism and Chirality in Organic Nano- and Microfilaments. ACS Nano 2021, 15 (4), 7249–7270. 10.1021/acsnano.1c00527. 33734664

[ref51] Castillo-Vallés M.; Folcia C. L.; Ortega J.; Etxebarria J.; Blanca Ros M. Self-assembly of bent-core amphiphiles joining the ethylene-oxide/lithium ion tandem. J. Mol. Liq. 2023, 381, 121825–121835. 10.1016/j.molliq.2023.121825.

[ref52] Matsunaga K.; Tajima M.; Yoshida Y. Thermal degradation of carboxylate-based polyurethane anionomers. J. Appl. Polym. Sci. 2006, 101, 573–579. 10.1002/app.23574.

[ref53] Espinosa T.; Sanes J.; Jiménez A.-E.; Bermúdez M.-D. Protic ammonium carboxylate ionic liquid lubricants of OFHC copper. Wear 2013, 303, 495–509. 10.1016/j.wear.2013.03.041.

[ref54] Nierengarten I.; Guerra S.; Holler M.; Nierengarten J.-F.; Deschenaux R. Building liquid crystals from the 5-fold symmetrical pillar[5]arene core. Chem. Commun. 2012, 48, 8072–8074. 10.1039/c2cc33746k. 22781925

[ref55] Shimura H.; Yoshio M.; Hoshino K.; Mukai T.; Ohno H.; Kato T. Noncovalent Approach to One-Dimensional Ion Conductors: Enhancement of Ionic Conductivities in Nanostructured Columnar Liquid Crystals. J. Am. Chem. Soc. 2008, 130 (5), 1759–1765. 10.1021/ja0775220. 18193872

[ref56] Kato T.; Yoshio M.; Ichikawa T.; Soberats B.; Ohno H.; Funahashi M. Transport of ions and electrons in nanostructured liquid crystals. Nat. Rev. Mater. 2017, 2, 17001–17021. 10.1038/natrevmats.2017.1.

[ref57] Conejo-Rodríguez V.; Cuerva C.; Schmidt R.; Bardají M.; Espinet P. Li^+^ and K^+^ ionic conductivity in ionic nematic liquid crystals based on 18-diaza-crown ether substituted with six decylalkoxy-p-cyanobiphenyl chains. J. Mater. Chem. C 2019, 7, 663–672. 10.1039/C8TC04898C.

[ref58] Kong S.; Wang X.; Bai L.; Song Y.; Meng F. Multi-arm ionic liquid crystals formed by pyridine-mesophase and copper phthalocyanine. J. Mol. Liq. 2019, 288, 111012–111020. 10.1016/j.molliq.2019.111012.

[ref59] Chen D.; Heberling M.-S.; Nakata M.; Hough L. E.; Maclennan J. E.; Glaser M. A.; Korblova E.; Walba D. M.; Watanabe J.; Clark N. A. Structure of the B4 Liquid Crystal Phase near a Glass Surface. ChemPhysChem 2012, 13, 155–159. 10.1002/cphc.201100589. 22162333

[ref60] Umadevi S.; Ganesh V.; Berchmans S. Liquid crystal (LC) monolayer on Indium Tin Oxide (ITO): structural and electrochemical characterization. RSC Adv. 2014, 4, 16409–16417. 10.1039/C4RA00556B.

[ref61] Concellón A.; Liang T.; Schenning A. P. H. J.; Serrano J. L.; Romero P.; Marcos M. Proton-conductive materials formed by coumarin photocrosslinked ionic liquid crystal dendrimers. J. Mater. Chem. C 2018, 6, 1000–1007. 10.1039/C7TC05009G.

[ref62] Ahn S.; Yamakawa S.; Akagi K. Liquid crystallinity-embodied imidazolium-based ionic liquids and their chiral mesophases induced by axially chiral tetra-substituted binaphthyl derivatives. J. Mater. Chem. C 2015, 3, 3960–3970. 10.1039/C4TC02968B.

[ref63] Martinez-Felipe A.; Zaton D.; Castillo-Vallés M.; Baldini A.; Pease J.; Leader N.; Aripin N. F. K.; Giacinti-Baschetti M.; Ros M. B. Bent-core liquid crystals joining the ethylene-oxide/lithium ion tandem: Ionic conductivity and dielectric response towards new electrolytes for energy applications. J. Mol. Liq. 2023, 390, 123100 10.1016/j.molliq.2023.123100.

[ref64] Lavoine N.; Bras J.; Saito T.; Isogai A. Optimization of preparation of thermally stable cellulose nanofibrils via heat-induced conversion of ionic bonds to amide bonds. J. Polym. Sci., Part A: Polym. Chem. 2017, 55, 1750–1756. 10.1002/pola.28541.

[ref65] Oechsle A. L.; Schöner T.; Geiger C.; Tu S.; Wang P.; Cubitt R.; Müller-Buschbaum P. Unraveling the Humidity Influence on the Electrical Properties of Ionic Liquid Posttreated Poly(3,4-ethylenedioxythiophene): Poly(styrenesulfonate) Films. Macromolecules 2023, 56, 9117–9126. 10.1021/acs.macromol.3c01842.

[ref66] De S.; Cramer C.; Schönhoff M. Humidity Dependence of the Ionic Conductivity of Polyelectrolyte Complexes. Macromolecules 2011, 44, 8936–8943. 10.1021/ma201949s.

[ref67] Oh M.; Lim S.-I.; Jang J.; Wi Y.; Yu D.; Hyeong J.; Kim S.; Kim W.; Ha M.; Jeong K.-U. Ionic Conductivity Switchable and Shape Changeable Smart Skins with Azobenzene-Based Ionic Reactive Mesogens. Adv. Funct. Mater. 2024, 34, 2307011–2307023. 10.1002/adfm.202307011.

[ref68] Kang N.; Li P.; Tan S.; Wang C. Azobenzene based inorganic salts for light modulated ionic conductivity in aqueous solution. Soft Matter 2019, 15, 7992–7995. 10.1039/C9SM01411J. 31502625

[ref69] Li Z.; Yuan X.; Feng Y.; Chen Y.; Zhao Y.; Wang H.; Xu Q.; Wang J. A reversible conductivity modulation of azobenzene-based ionic liquids in aqueous solutions using UV/vis light. Phys. Chem. Chem. Phys. 2018, 20, 12808–12816. 10.1039/c8cp01617h. 29700535

[ref70] Hada M.; Yamaguchi D.; Ishikawa T.; Sawa T.; Tsuruta K.; Ishikawa K.; Koshihara S.-Y.; Hayashi Y.; Kato T. Ultrafast isomerization-induced cooperative motions to higher molecular orientation in smectic liquid-crystalline azobenzene molecules. Nat. Commun. 2019, 10, 4159 10.1038/s41467-019-12116-6. 31519876 PMC6744564

[ref71] Trisovic N.; Antanasijevic J.; Toth-Katona T.; Kohout M.; Salamonczyk M.; Sprunt S.; Jakli A.; Fodor-Csorba K. Azo-containing asymmetric bent-core liquid crystals with modulated smectic phases. RSC Adv. 2015, 5, 64886–64891. 10.1039/C5RA09764A.

[ref72] Mai Y.; Eisenberg A. Selective Localization of Preformed Nanoparticles in Morphologically Controllable Block Copolymer Aggregates in Solution. Acc. Chem. Res. 2012, 45 (10), 1657–1666. 10.1021/ar2003144. 22839780

[ref73] Yu Y.; Eisenberg A. Control of Morphology through Polymer–Solvent Interactions in Crew-Cut Aggregates of Amphiphilic Block Copolymers. Am. Chem. Soc. 1997, 119 (35), 8383–8384. 10.1021/ja9709740.

[ref74] Chou S.-H.; Wu D. T.; Tsao H.-K.; Sheng Y.-J. Morphology and internal structure control of rod-coil copolymer aggregates by mixed selective solvents. Soft Matter 2011, 7, 9119–9129. 10.1039/c1sm05808h.

[ref75] Cheng X.; Miao T.; Yin L.; Ji Y.; Li Y.; Zhang Z.; Zhang W.; Zhu X. In Situ Controlled Construction of a Hierarchical Supramolecular Chiral Liquid Crystalline Polymer Assembly. Angew. Chem., Int. Ed. 2020, 59, 9669–9677. 10.1002/anie.202001657. 32181944

[ref76] Gu K.; Yang W.; Wen T.; Wang Q.; Zhang W.; Han M.; Shen Z.; Fan X.; Ho R.-M. Co-assembled twisted superstructures formed by disc-bent core amphiphiles. Giant 2022, 9, 100087 10.1016/j.giant.2021.100087.

[ref77] Lin S.-C.; Ho R.-M.; Chang C. Y.; Hsu C.-S. Hierarchical Superstructures with Control of Helicity from the Self-Assembly of Chiral Bent-Core Molecules. Chem. - Eur. J. 2012, 18, 9091–9098. 10.1002/chem.201200057. 22689474

