# Supplementary material for: Ionic Bent-Core Pillar[n]arenes: From Liquid Crystals to Nanoaggregates and Functional Applications
Source: Chem Mater. 2024 Oct 2;36(19):9793–805. doi: 10.1021/acs.chemmater.4c01952 (PMC11468781; doi:10.1021/acs.chemmater.4c01952)
Supplement: Supplementary file 1 — cm4c01952_si_001.pdf [file cm4c01952_si_001.pdf]

Ionic bent-core pillar[n]arenes: from liquid crystals to nanoaggregates and functional applications.

Iván Marín[a,b], Martín Castillo-Vallés[a,b], Rosa I. Merino[a,c], César L. Folcia[d], Joaquín Barberá[a,b], M. Blanca Ros[a,b,*] and José L. Serrano[a,b,*]

[a] Instituto de Nanociencia y Materiales de Aragón (INMA), CSIC-Universidad de Zaragoza, 50009 Zaragoza, Spain.

[b] Departamento de Química Orgánica, Facultad de Ciencias, Universidad de Zaragoza, 50009 Zaragoza, Spain.

[c] Departamento de Física de la Materia Condensada, Facultad de Ciencias, CSIC-Universidad de Zaragoza, 50009 Zaragoza, Spain.

[d] Departamento de Física, Facultad de Ciencia y Tecnología, Universidad del País Vasco, E-48080 Bilbao, Spain

** Author for correspondence: joseluis@unizar.es bros@unizar.es*

Table of Contents

1. Materials and Characterization techniques S2

2. Experimental procedures S3

2.1 General procedure for the preparation of ionic complexes S3

2.2 General procedure for the preparation of aggregates S6

2.3 Irradiation experiments S6

3. Supplementary figures S7

3.1 NMR spectra S7

3.2 FTIR spectra S13

3.3 POM textures S14

3.4 DSC thermograms S15

3.5 X-ray diffractograms S16

3.6 EIS measurements S19

3.7 UV-vis spectra S20

3.8 TEM Images S21

1. MATERIALS AND CHARACTERIZATION TECHNIQUES

All reagents were purchased from Aldrich and used without further purification. Anhydrous CH_2Cl_2 and THF were purchased from Scharlab and dried by using a solvent purification system.
^{1}H-NMR and **^{13}C-NMR** spectra were acquired on a Bruker AV400 spectrometer. The experiments were performed at room temperature in different deuterated solvents ($CDCl_3$ CD_2Cl_2 or DMSO-d_6). Chemical shifts are given in ppm relative to TMS and the solvent residual peak was used as the internal standard.
Infrared spectra were recorded on a Bruker Vertex 70 FT-IR spectrometer. The samples were prepared on KBr pellets with a concentration of the product of 1-2% (w/w).
Mass spectra were obtained on a MICROFLEX Bruker (MALDI$^+$) spectrometer with a dithranol matrix.
Mesogenic behavior was investigated by polarized-light optical microscopy (POM) using an Olympus BH-2 polarizing microscope fitted with a Linkam THMS600 hot stage. Thermogravimetric analysis (TGA) was performed using a Q5000IR from TA instruments at heating rate of 10 °C min^{-1} under a nitrogen atmosphere. Thermal transitions were determined by differential scanning calorimetry (DSC) using a DSC Q2000 from TA instruments with powdered samples (2–5 mg) sealed in aluminum pans. Glass transition temperatures (Tg) were determined at the half height of the baseline jump, and first order transition temperatures were read at the maximum of the corresponding peak.
X-ray diffraction diagrams were recorded using a Stoe Stadivari goniometer equipped with a Genix3D microfocus generator (Xenocs) and a Dectris Pilatus 100K detector. Temperature control was achieved using a nitrogen-gas Cryostream controller (Oxford Cryosystems) allowing for a temperature control of about 0.1 ºC. Lindemann capillaries of diameter 0.6 mm were utilized. Monochromatic CuKα radiation (λ = 1.5418 Å) was used. The exposure time was 2 minutes.
UV-Vis absorption spectra were recorded on an ATI-Unicam UV4-200 spectrophotometer.
Electrochemical impedance spectroscopy (EIS) was recorded with a Frequency Response Analyzer, Solarton SI1260A from AMETEK in the frequency range from 1 Hz to 1 MHz (amplitude of the applied voltage: 50 mV). The temperature of the sample was controlled with Linkam THMS600 hot stage. The conductivities were studied as a function of temperature between 30°C and isotrope temperature at different intervals in the heating and in the cooling runs. At each step the temperature was hold until equilibration before measurement. On average the net heating and cooling rates were between 1 and 3°C/min. For the preparation of the cells for ionic conductivities, the appropriate amount of the ionic compound was placed into an ITO electrode that was sandwiched with another ITO electrode controlling the thickness by using glass spacers (10-20 µm). The cell was heated up to a few degrees above the melting point of the liquid crystal and the cell was pressed to obtain the thin film. The measured impedance spectra were plotted in complex plane plots, imaginary (Z’’) versus real (Z’) component. They consist of a

high frequency arc due to the sample electrical response, more or less overlapped with the low frequency electrode contribution. This assignment was made according to their equivalent capacitances. The sample resistance (R_b) was estimated from the minimum of the –Z” vs Z′ at the low frequency side of the high frequency arc (sample contribution). The conductivities, assigned to proton conductivities, σ ($S·cm^{-1}$) were calculated with the formula: $\sigma = d / (R_b \cdot A)$), where d (cm) is the thickness of the film, A (cm^2) is the area of the film and R_b (Ω) is the resistance of the sample.
After the preparation of the cell, a random orientation of the mesophase was observed between electrodes. Samples were mechanically sheared within the cell (in order to obtain an alignment of the molecules) at isotropic temperature and then slowly cool down to room temperature (0.05 $^{o}C·min^{-1}$).
Microscopy (TEM) analysis was performed using a FEI Tecnai T20 microscope (FEI Company, Waltham, MA, USA) operating at 200 kV. TEM samples were prepared adding 10 μL of each self-assembly dispersion at an approximately 1.0 mg mL^{-1} concentration on a continuous carbon film-copper grid, and the excess was removed by capillarity using filter paper. Then, the grids were stained with uranyl acetate (1% aqueous solution), removing the excess again by capillarity using filter paper.

2. EXPERIMENTAL PROCEDURES

2.1 General procedure for the preparation of ionic complexes

The synthesis of different acids and pillar[5]arene **P5N10** was carried out following antecedent papers. Ionic dendrimers were prepared following the previously described methodology. A solution of the corresponding acid in dry tetrahydrofuran was added to a solution of the pillar[5]arene **P5N10**, in approximately 1:1 (primary amine groups: carboxylic acid groups) stoichiometry. The mixture was ultrasonicated for 60 min, and then it was slowly evaporated at room temperature and dried in vacuum at 40°C until the weight remained constant.

- COMPOUNDS B1 4-8-P5N10 and B1 10-8-P5N10

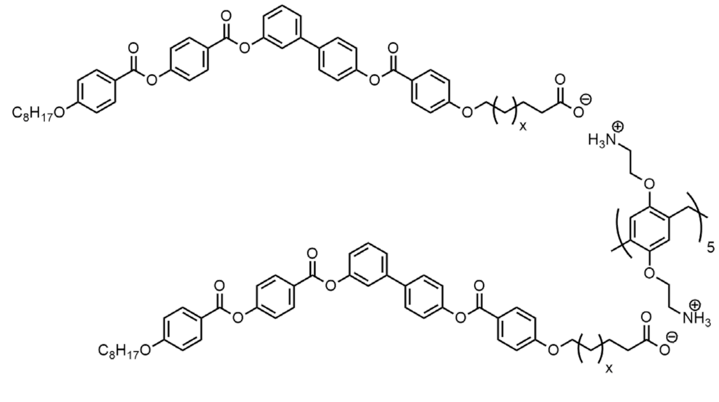

Compound B1 4-8-P5N10 *(x: 1)*

^{1}H-NMR ($CDCl_3$, 298K, 400 MHz, δ, ppm): 8.28 (d, J=8.2 Hz, 20H), 8.13 (m, 40H), 7.62 (d, J=9.0 Hz, 20H), 7.46 (m, 30H), 7.36 (d, J=8.5 Hz, 20H), 7.20 (m, 20H), 6.95

(m, 40H), 5.85 (s, 30H), 4.04 (m, 40H), 3.71 (s, 10H), 3.40 (s, 10H), 2.36 (m, 20H), 1.83 (m, 40H), 1.52-1.23 (m, 120H), 0.89 (t, J=6.9 Hz, 30H).

^{13}C-NMR (CDCl$_3$, 298K, 100 MHz, δ, ppm): 179.38, 165.01, 164.61, 164.46, 163.96, 163.50, 155.54, 151.45, 150.91, 142.16, 137.82, 132.56, 132.42, 131.95, 129.99, 128.33, 126.96, 124.78, 122.30, 122.26, 121.66, 121.08, 120.48, 114.55, 114.40, 110.14, 68.53, 68.02, 35.76, 31.94, 29.47, 29.36, 29.23, 28.92, 26.12, 22.80, 22.38, 14.24.

FTIR (KBr, ν: cm^{-1}): 3437 (-NH$_3^+$), 2919 (=C-H), 2848 (-C-H), 1734 (C=O), 1606, 1580 (C=C), 1258, 1151 (C-O).

Compound B1 10-8-P5N10 ***(x: 7)***

^{1}H-NMR (CDCl$_3$, 298K, 400 MHz, δ, ppm): 8.31 (d, J=8.8 Hz, 20H), 8.16 (m, 40H), 7.65 (d, J=8.7 Hz, 20H), 7.48 (m, 30H), 7.38 (d, J=8.8 Hz, 20H), 7.29 (d, J=8.6 Hz, 20H), 7.22 (m, 10H), 6.98 (m, 40H), 4.05 (m, 40H), 2.34 (t, J=7.5 Hz, 20H), 1.82 (m, 40H), 1.63 (m, 20H), 1.53-1.20 (m, 200H), 0.90 (t, J=6.9 Hz, 30H).

^{13}C-NMR (CDCl$_3$, 298K, 100 MHz, δ, ppm): 179.03, 165.10, 164.66, 164.50, 163.98, 163.73, 155.57, 151.46, 150.98, 142.29, 137.89, 132.58, 132.47, 131.99, 130.01, 128.39, 127.00, 124.84, 122.33, 122.28, 121.58, 121.09, 120.71, 120.56, 114.57, 114.46, 68.55, 68.46, 35.30, 31.95, 29.67, 29.60, 29.51, 29.48, 29.37, 29.25, 29.24, 26.14, 25.48, 22.81, 14.25.

FTIR (KBr, ν: cm^{-1}): 3441 (-NH$_3^+$), 2921 (=C-H), 2856 (-C-H), 1735 (C=O), 1604, 1509 (C=C), 1256, 1163 (C-O).

- COMPOUNDS Bi 4-8-P5N10 and Bi 10-8-P5N10

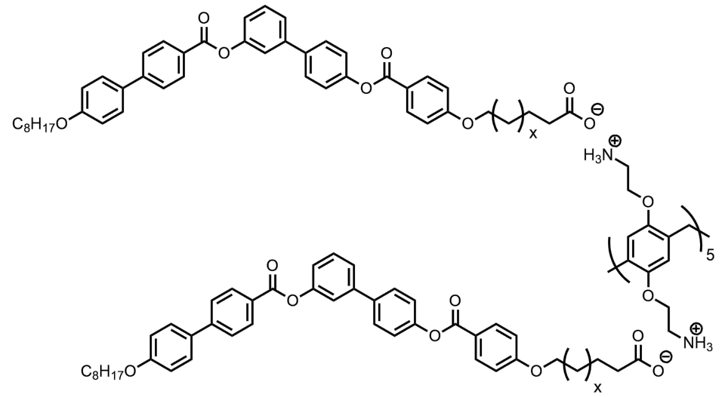

Compound Bi 4-8-P5N10 ***(x: 1)***

^{1}H-NMR (CDCl$_3$, 298K, 400 MHz, δ, ppm): 8.23 (d, J=8.4 Hz, 20H), 8.10 (d, J=8.7 Hz, 20H), 7.67 (d, J= 8.5 Hz, 20H), 7.59 (m, 40H), 7.45 (m, 30H), 7.22 (m, 30H), 6.99 (d, J=8.8 Hz, 20H), 6.90 (d, J=8.7 Hz, 20H), 5.19 (s, 30H), 4.00 (t, J=6.5 Hz, 40H), 3.74 (m ,20H), 3.43 (s, 10H), 2.99 (m, 20H), 2.37 (m, 20H), 1.81 (m, 60H), 1.54-1.17 (m, 120H), 0.89 (t, J=7.0 Hz, 30H).

^{13}C-NMR ($CDCl_3$, 298K, 100 MHz, δ, ppm): 165.30, 163.54, 159.86, 151.74, 151.08, 146.30, 142.32, 138.05, 132.51, 132.24, 131.19, 130.93, 129.97, 128.57, 128.41, 127.74, 126.81, 124.73, 122.32, 122.03, 120.80, 120.66, 118.28, 115.27, 114.53, 68.46, 68.43, 67.92, 33.44, 31.99, 29.57, 29.53, 29.45, 29.40, 28.70, 26.24, 22.81, 21.70, 14.23.

FTIR (KBr, ν: cm^{-1}): 3439 ($-NH_3^+$), 2931 (=C-H), 2854 (-C-H), 1735 (C=O), 1604 (C=C), 1258 (C-O), 1167(C-O).

Compound Bi 10-8-P5N10 ***(x: 7)***

^{1}H-NMR ($CDCl_3$, 298K, 400 MHz, δ, ppm): 8.26 (d, J=8.4 Hz, 20H), 8.16 (d, J=8.9 Hz, 20H), 7.71 (d, J=8.4 Hz, 20H), 7.66 (d, J=8.6 Hz, 20H), 7.61 (d, J=8.7 Hz, 20H), 7.51 (d, J=4.9 Hz, 20H), 7.46 (s, 10H), 7.26 (d, J=8.6 Hz, 20H), 7.00 (m, 40H), 4.04 (m, 40H), 2.36 (t, J=7.6 Hz, 20H), 1.82 (q, J=7.7 Hz, 40H), 1.70-1.23 (m, 200H), 0.90 (t, J=6.9 Hz, 30H).

^{13}C-NMR ($CDCl_3$, 298K, 100 MHz, δ, ppm): 174.05, 165.30, 165.09, 163.74, 159.76, 151.60, 150.99, 146.19, 142.28, 137.96, 132.46, 132.12, 132.10, 130.90, 129.96, 128.54, 128.40, 127.61, 126.76, 124.74, 122.32, 121.62, 120.76, 120.63, 115.15, 114.47, 68.47, 68.33, 34.53, 34.25, 31.97, 29.61, 29.51, 29.39, 29.27, 29.24, 26.20, 25.91, 25.12, 22.81, 14.25.

FTIR (KBr, ν: cm^{-1}): 3426 ($-NH_3^+$), 2921 (=C-H), 2851 (-C-H), 1732 (C=O), 1704 (C=O dimeric), 1604 (C=C), 1251 (C-O), 1171 (C-O).

- COMPOUNDS Bazo 4-8-P5N10 and Bazo 10-8-P5N10

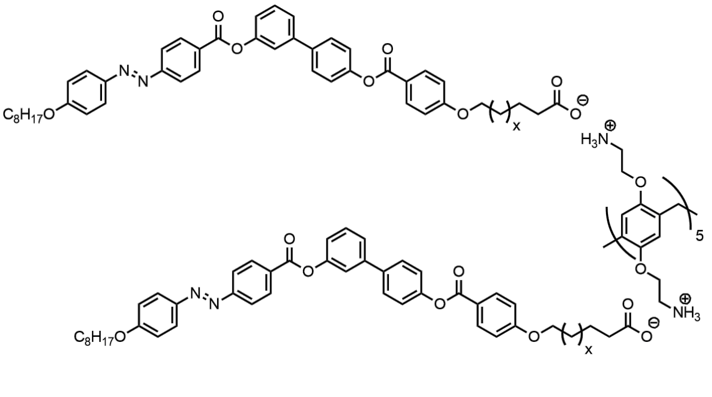

Compound Bazo 4-8-P5N10 ***(x: 1)***

^{1}H-NMR ($CDCl_3$, 298K, 400 MHz, δ, ppm): 8.33 (d, J=8.3 Hz, 20H), 8.12 (d, J=7.6 Hz, 20H), 7.95 (m, 40H), 7.62 (d, J=8.2 Hz, 20H), 7.47 (m, 30H), 7.24 (m, 20H), 7.01 (d, J=8.9 Hz, 20H), 6.93 (d, J=8.4 Hz, 20H), 4.99 (s, 30H), 4.05 (m, 40H), 3.75 (m, 20H), 3.43 (s, 10H), 2.41 (m, 20H), 1.83 (m, 40H), 1.54-1.23 (m, 120H), 0.90 (t, J=6.9 Hz, 30H).

^{13}C-NMR (CDCl$_3$, 298K, 100 MHz, δ, ppm): 178.22, 165.00, 164.88, 163.49, 162.62, 155.97, 151.48, 150.94, 147.01, 142.24, 137.87, 132.46, 131.38, 130.52, 130.01, 128.37, 125.46, 124.84, 122.69, 122.31, 121.75, 120.53, 114.97, 114.42, 68.61, 67.97, 39.83, 34.91, 31.96, 29.50, 29.38, 29.32, 28.83, 26.16, 22.81, 22.12, 14.26.

FTIR (KBr, ν: cm^{-1}): 3441 (-NH_3^+), 2921 (=C-H), 2841 (-C-H), 1732 (C=O), 1604 (C=C), 1251 (C-O).

Compound Bazo 10-8-P5N10 ***(x: 7)***

^{1}H-NMR (CDCl$_3$, 298K, 400 MHz, δ, ppm): 8.36 (d, J=8.3 Hz, 20H), 8.16 (d, J=7.6 Hz, 20H), 7.98 (m, 40H), 7.66 (d, J=8.2 Hz, 20H), 7.50 (m, 30H), 7.29 (d, J=8.9 Hz, 20H), 7.00 (m, 40H), 4.05 (m, 40H), 3.87 (m, 30H), 2.35 (t, J=7.5 Hz, 20H), 1.82 (m, 40H), 1.64 (m, 20H), 1.54-1.23 (m, 200H), 0.90 (t, J=6.9 Hz, 30H).

^{13}C-NMR (CDCl$_3$, 298K, 100 MHz, δ, ppm): 178.46, 165.10, 164.90, 163.74, 162.63, 156.01, 152.33, 151.50, 151.39, 151.00, 150.97, 147.03, 137.90, 137.35, 132.59, 132.34, 131.49, 131.28, 130.54, 128.59, 128.22, 125.61, 125.30, 122.80, 122.55, 122.35, 121.95, 121.59, 120.68, 115.10, 114.88, 114.57, 114.37, 68.63, 68.46, 65.33, 34.10, 31.96, 29.61, 29.58, 29.49, 29.37, 29.23, 26.15, 24.92, 22.81, 13.67.

FTIR (KBr, ν: cm^{-1}): 3427 (-NH_3^+), 2921 (=C-H), 2851 (-C-H), 1730 (C=O), 1604 (C=C), 1253 (C-O).

2.2 General procedure for the preparation of aggregates

For the preparation of the self-assemblies, a solution of 5 mg/mL of the amphiphilic ionic bent-core pillar[5]arene in THF was prepared, and Milli-Q water was gradually added while self-assembly was followed by measuring the turbidity in UV. When a critical water content was reached, a high increase in turbidity happened, indicating that the self-assembling process took place. Once turbidity reached an almost constant value, the mixture was dialyzed against water to remove the organic solvent using a Spectra/Por dialysis membrane (MWCO 1000) for 3 days. Water suspensions of the aggregates with a concentration around 2 mg/mL were obtained.

2.3 Irradiation experiments

Cells containing the materials **Bazo 10-8-P5N10** and **Bazo 4-8-P5N10** were irradiated with a led of 325 nm with an intensity of 300 mW/cm^2 at room temperature for 5 min.

3. SUPPLEMENTARY FIGURES

3.1 NMR Spectra

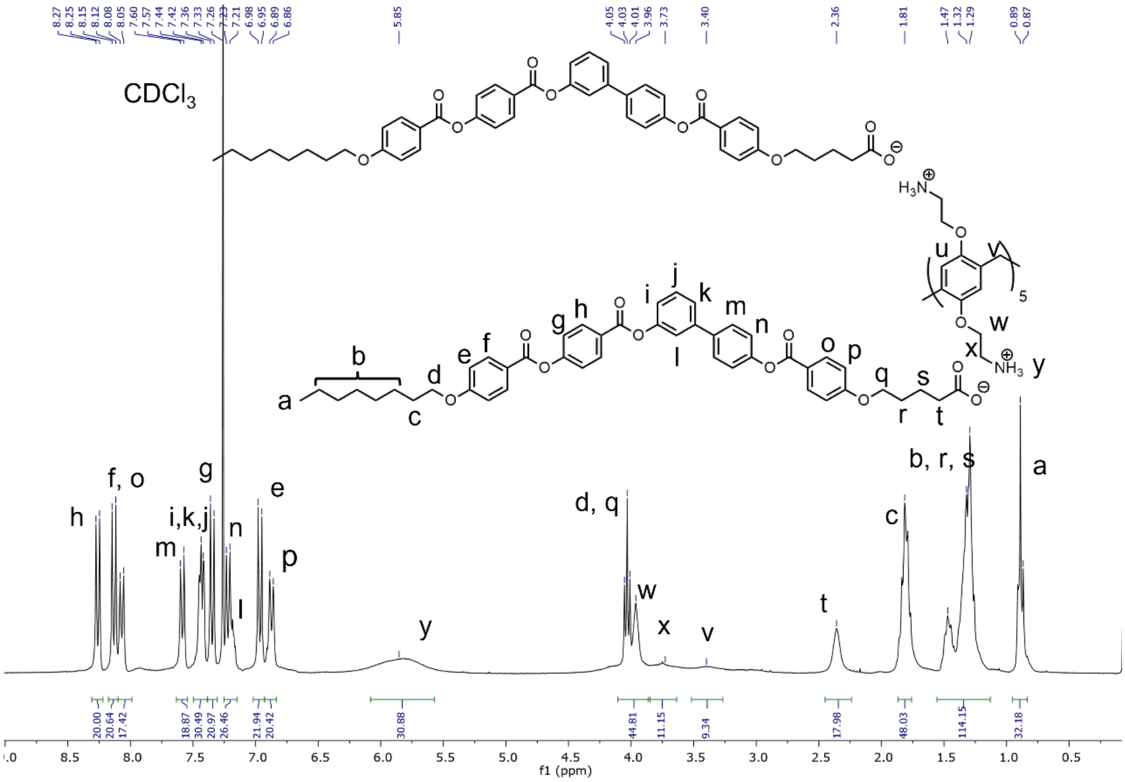

Figure S1. *¹H-NMR spectrum of* ***B1 4-8-P5N10*** *CDCl₃, 298K, 400 MHz.*

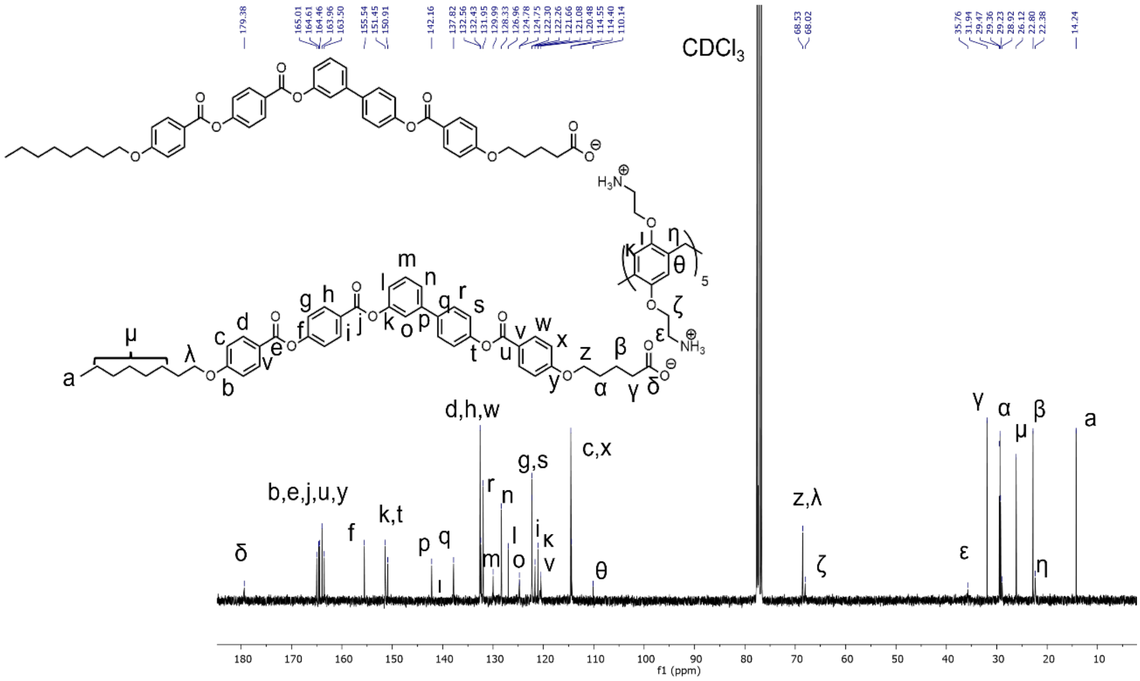

Figure S2. *¹³C-NMR spectrum of* ***B1 4-8-P5N10*** *CDCl₃, 298K, 100 MHz.*

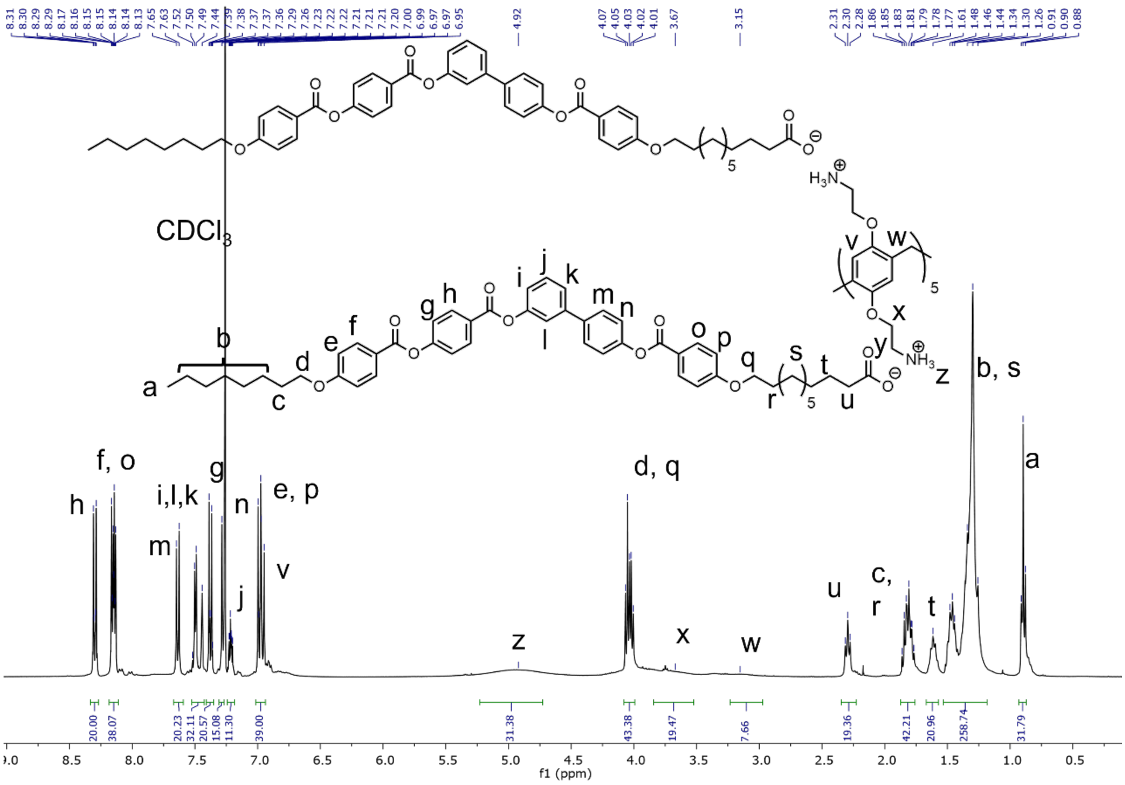

Figure S3. *^{1}H-NMR spectrum of* ***B1 10-8-P5N10*** *$CDCl_3$, 298K, 400 MHz.*

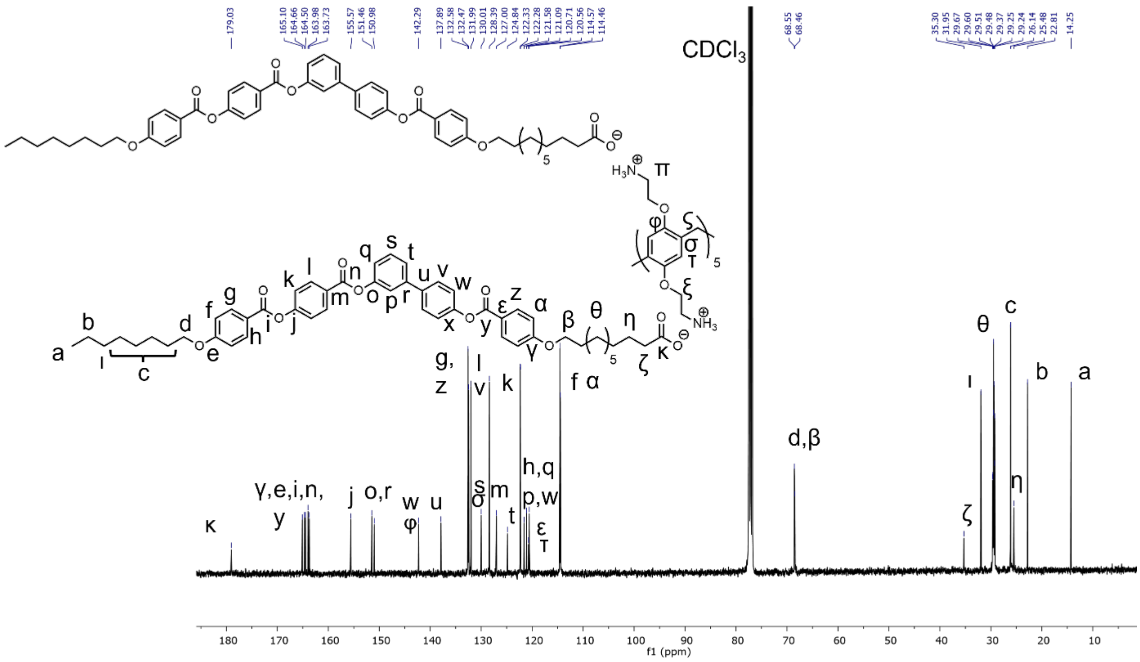

Figure S4. *^{13}C-NMR spectrum of* ***B1 10-8-P5N10*** *$CDCl_3$, 298K, 100 MHz.*

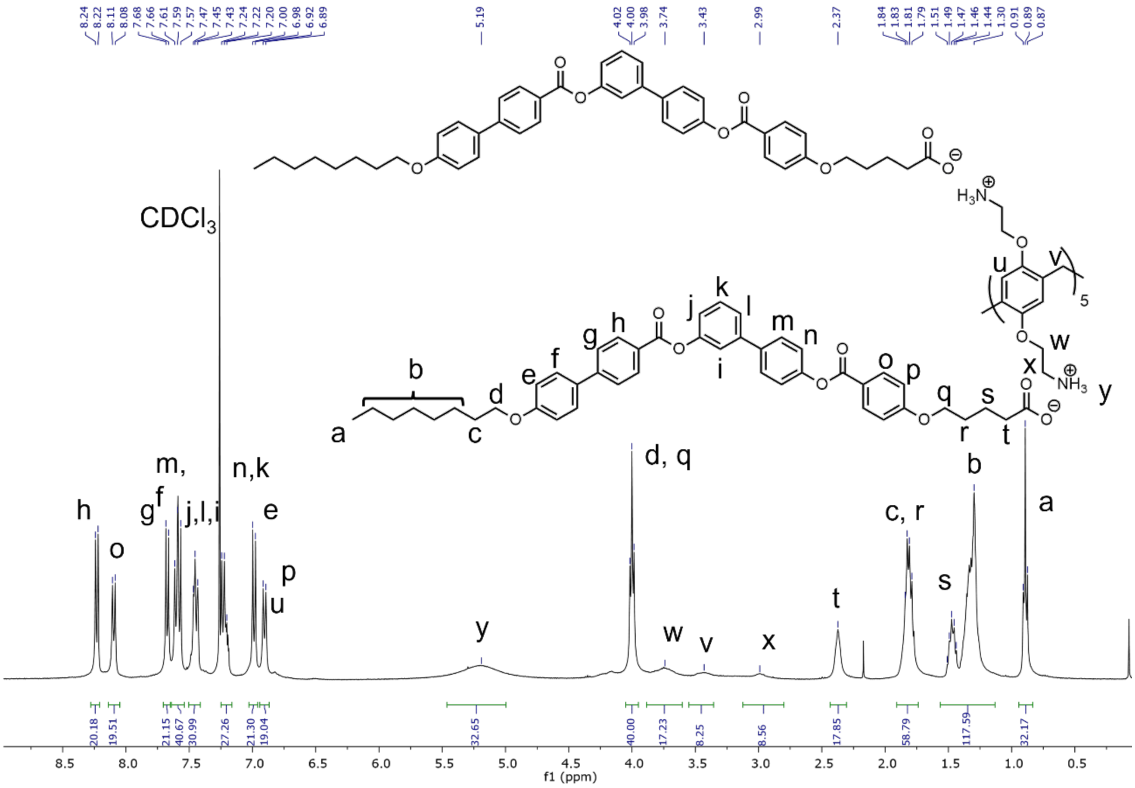

__Figure S5__. 1H-NMR spectrum of __Bi 4-8-P5N10__ CDCl3, 298K, 400 MHz.

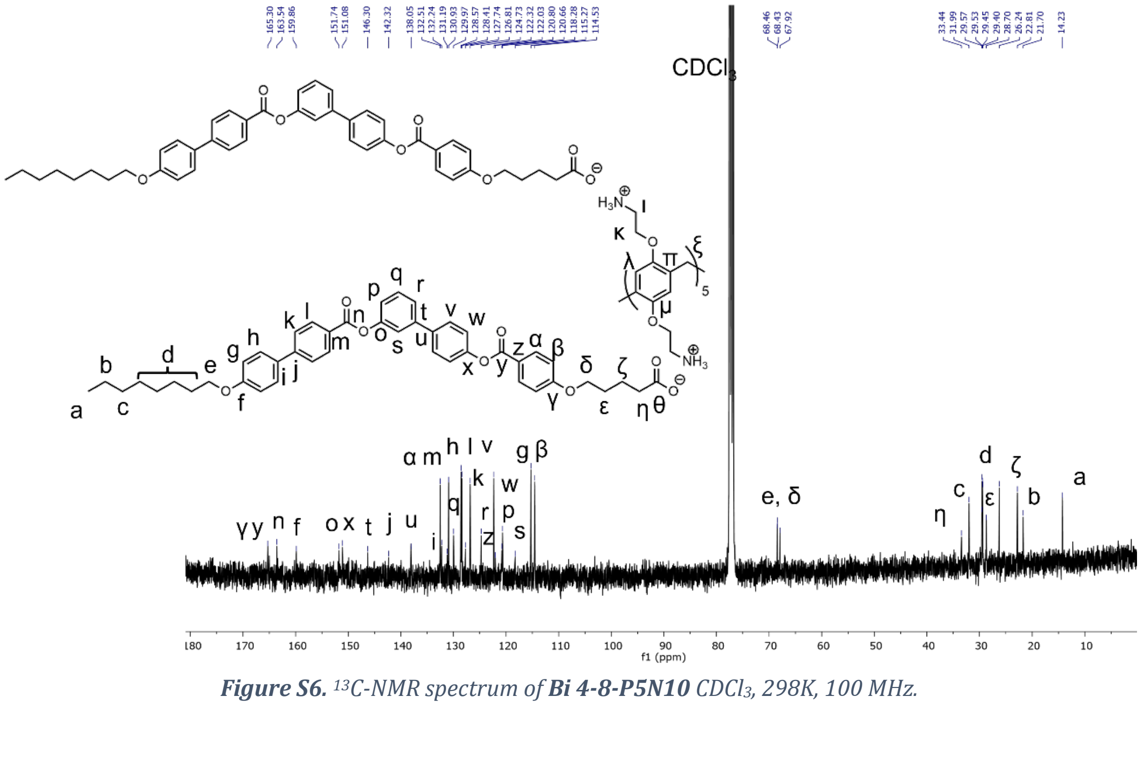

__Figure S6.__ 13C-NMR spectrum of __Bi 4-8-P5N10__ CDCl3, 298K, 100 MHz.

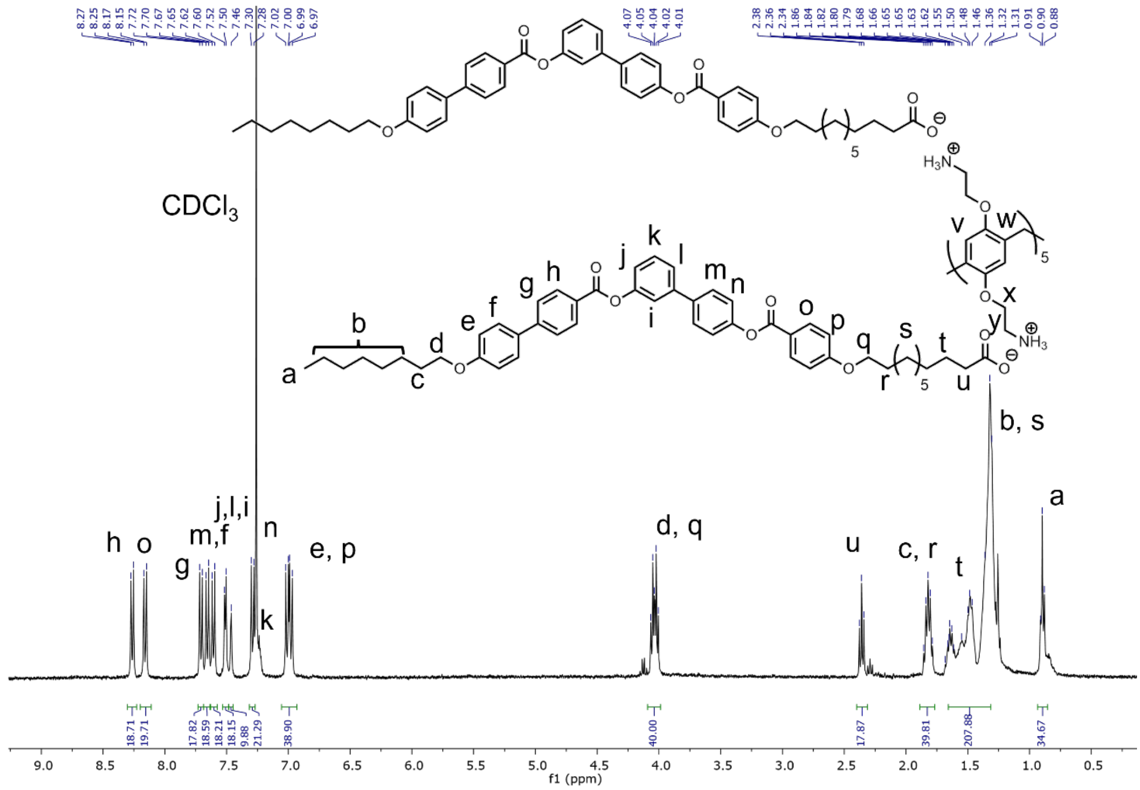

Figure S7. *^{1}H-NMR spectrum of* ***Bi 10-8-P5N10*** *CDCl$_3$, 298K, 400 MHz.*

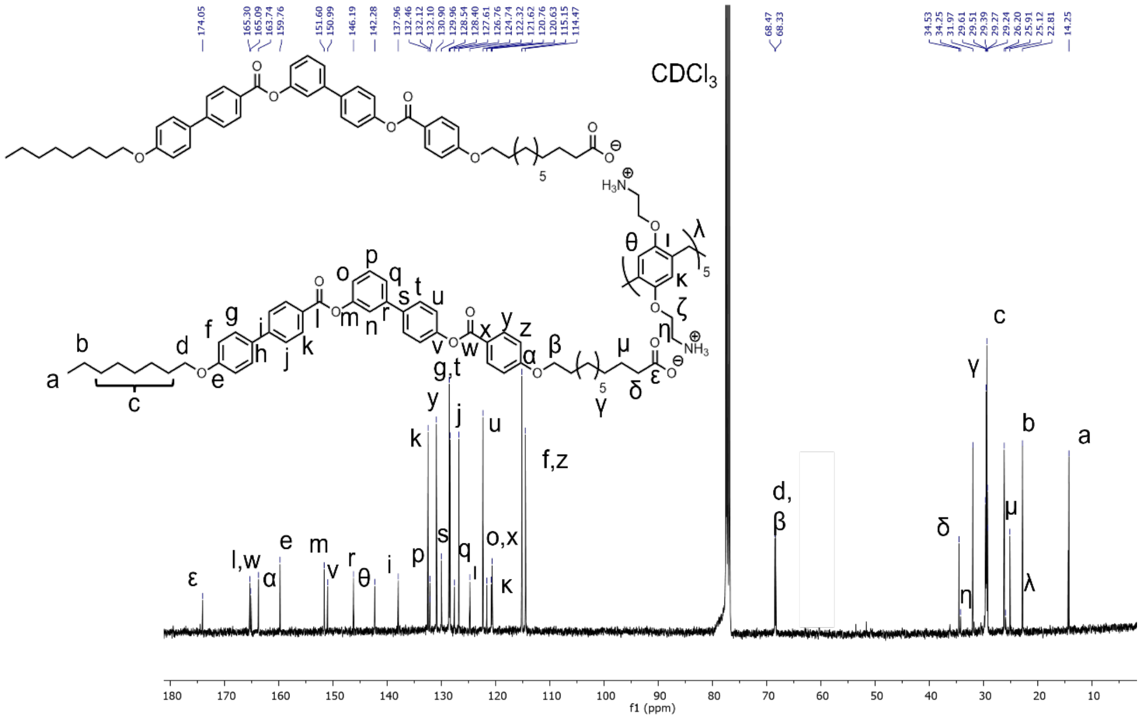

Figure S8. *^{13}C-NMR spectrum of* ***Bi 10-8-P5N10*** *CDCl$_3$, 298K, 100 MHz.*

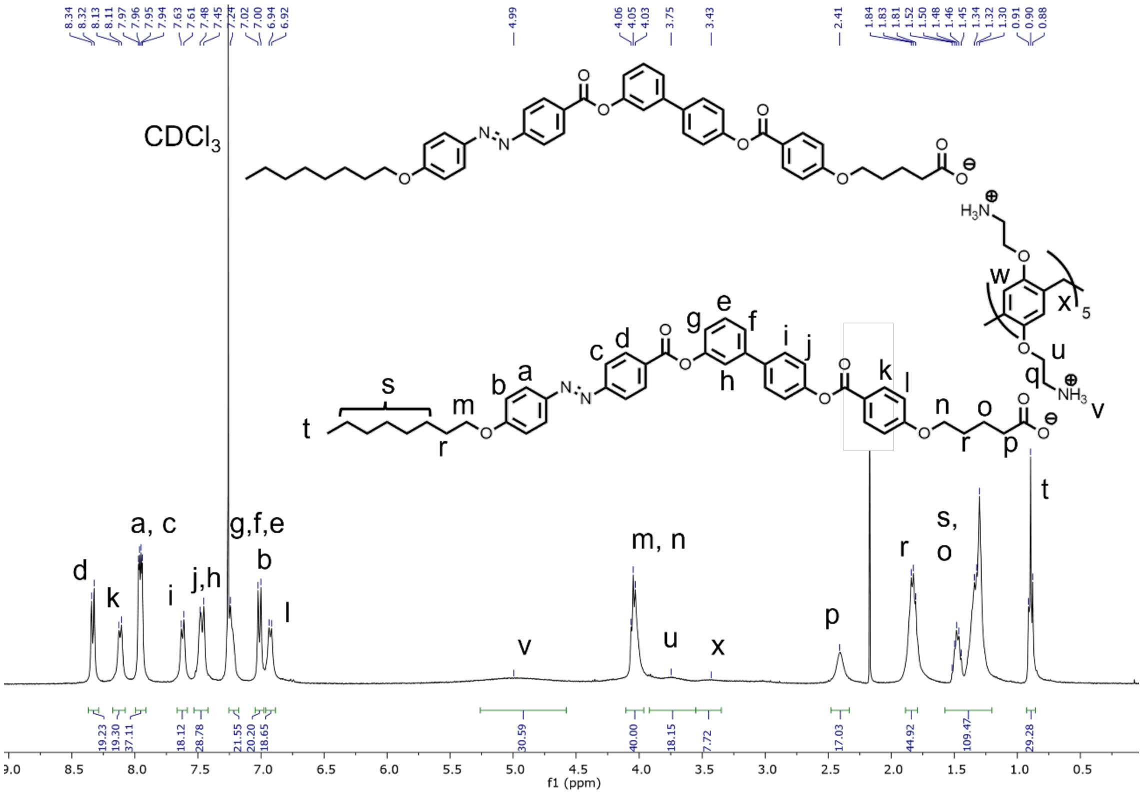

Figure S9. *^{1}H-NMR spectrum **Bazo 4-8-P5N10** $CDCl_3$, 298K, 400 MHz.*

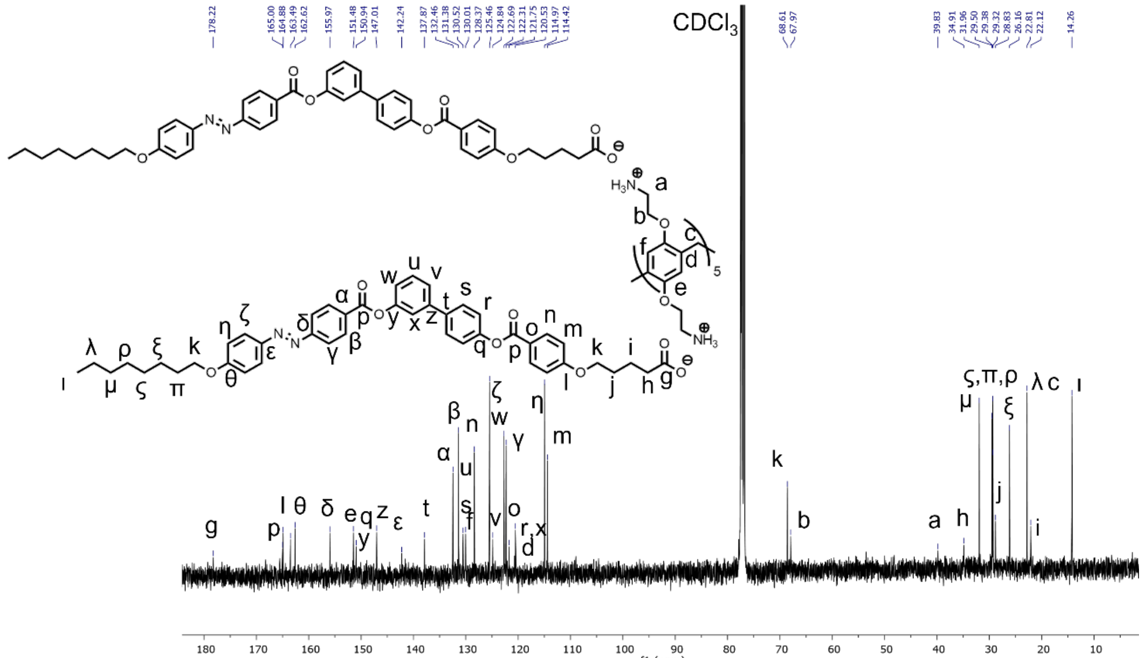

Figure S10. *^{13}C-NMR spectrum of **Bazo 4-8-P5N10** $CDCl_3$, 298K, 100 MHz.*

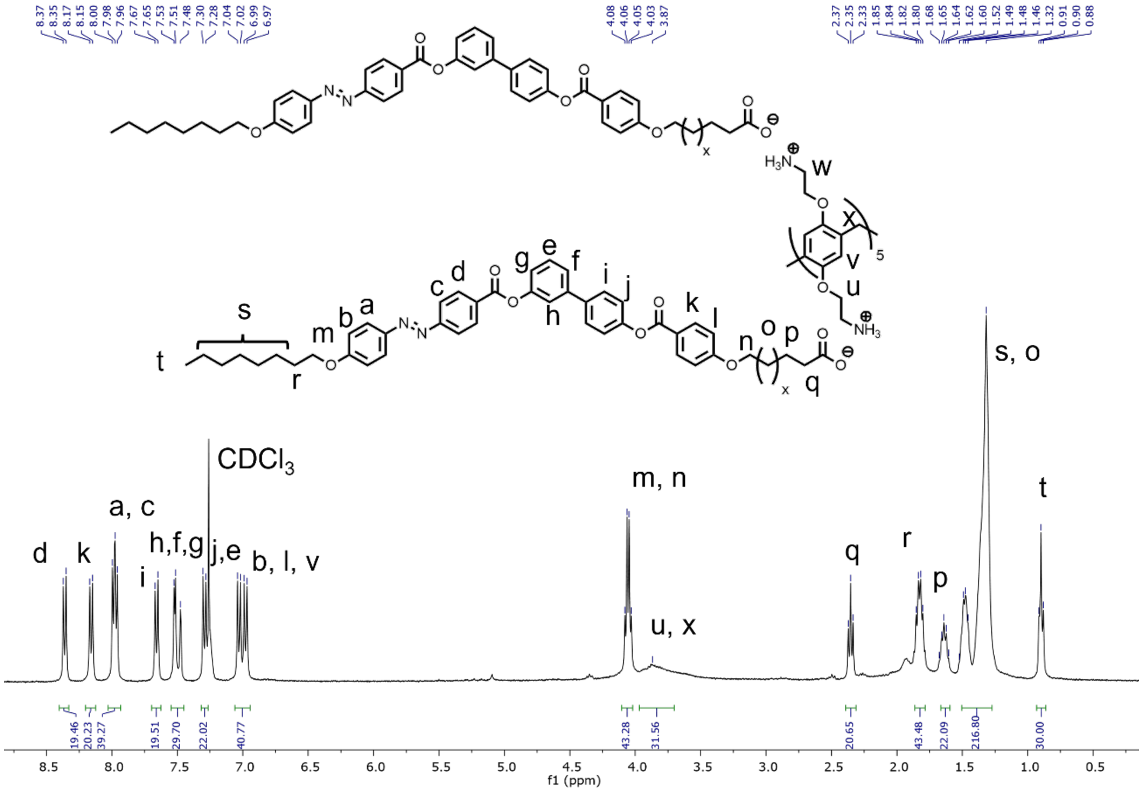

__Figure S11__. ^{1}H-NMR spectrum of __Bazo 10-8-P5N10__ CDCl₃, 298K, 400 MHz.

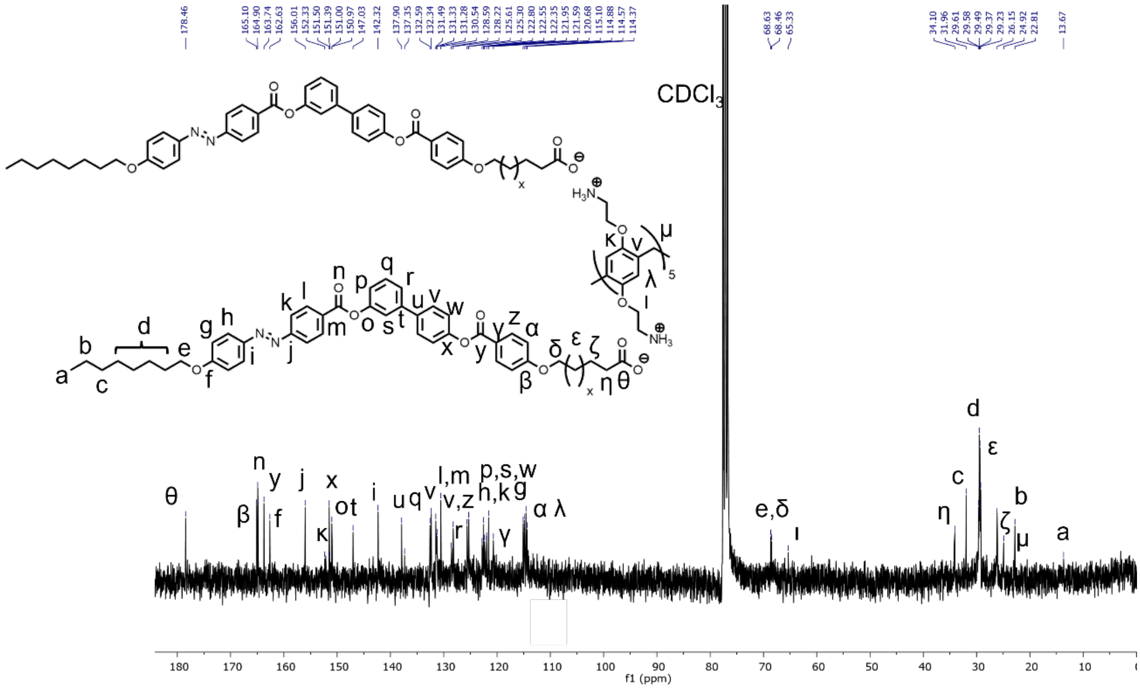

__Figure S12.__ ^{13}C-NMR spectrum of __Bazo 10-8-P5N10__ CDCl₃, 298K, 100 MHz.

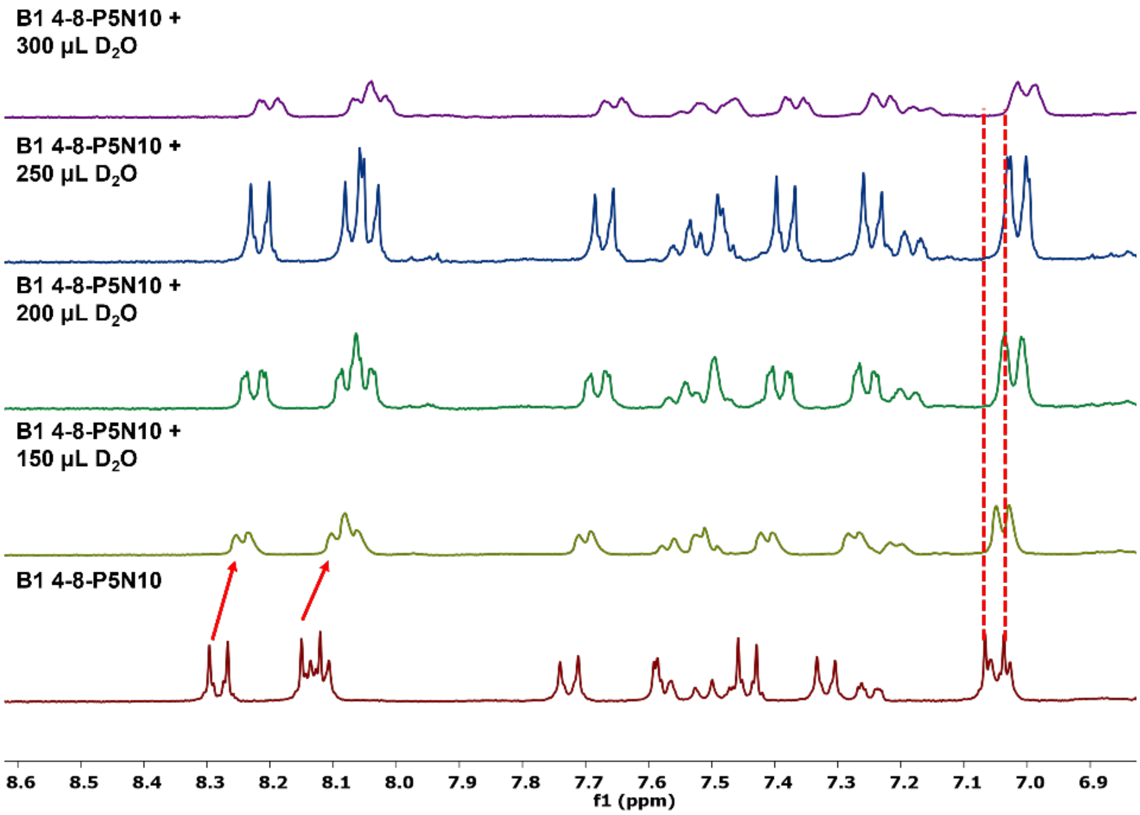

Figure S13. *Self-assembly process followed by ^{1}H-NMR of* ***B1 4-8-P5N10*** *adding D_2O aliquots.*

3.2 FT-IR spectra

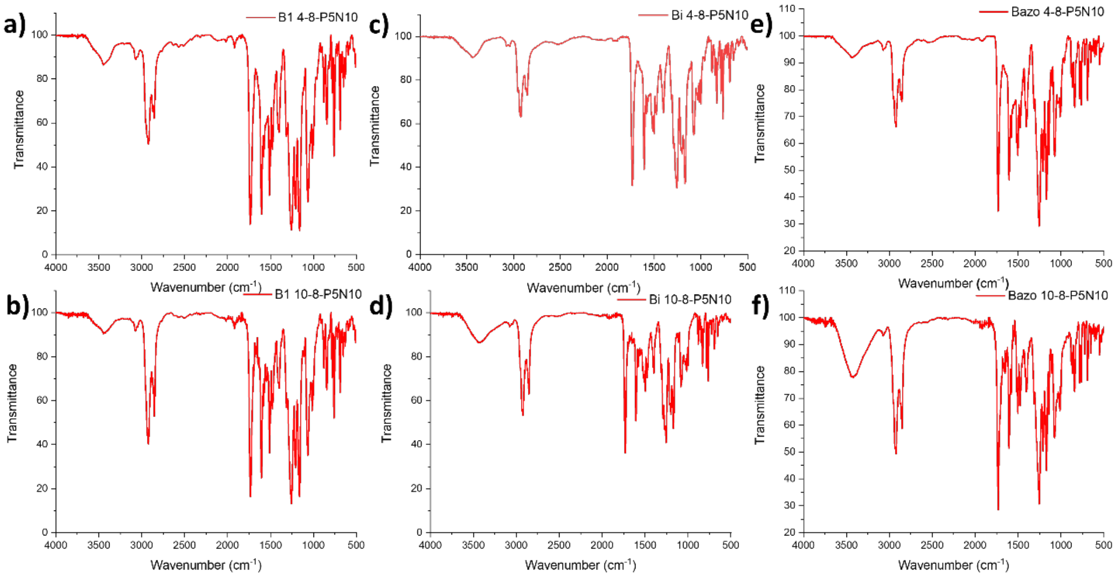

Figure S14*. FT-IR spectra in KBr of: a)* ***B1 4-8-P5N10****, b)* ***B1 10-8-P5N10****, c)* ***Bi 4-8-P5N10****, d)* ***Bi 10-8-P5N10****, e)* ***Bazo 4-8-P5N10*** *f)* ***Bazo 10-8-P5N10.***

3.3 POM textures

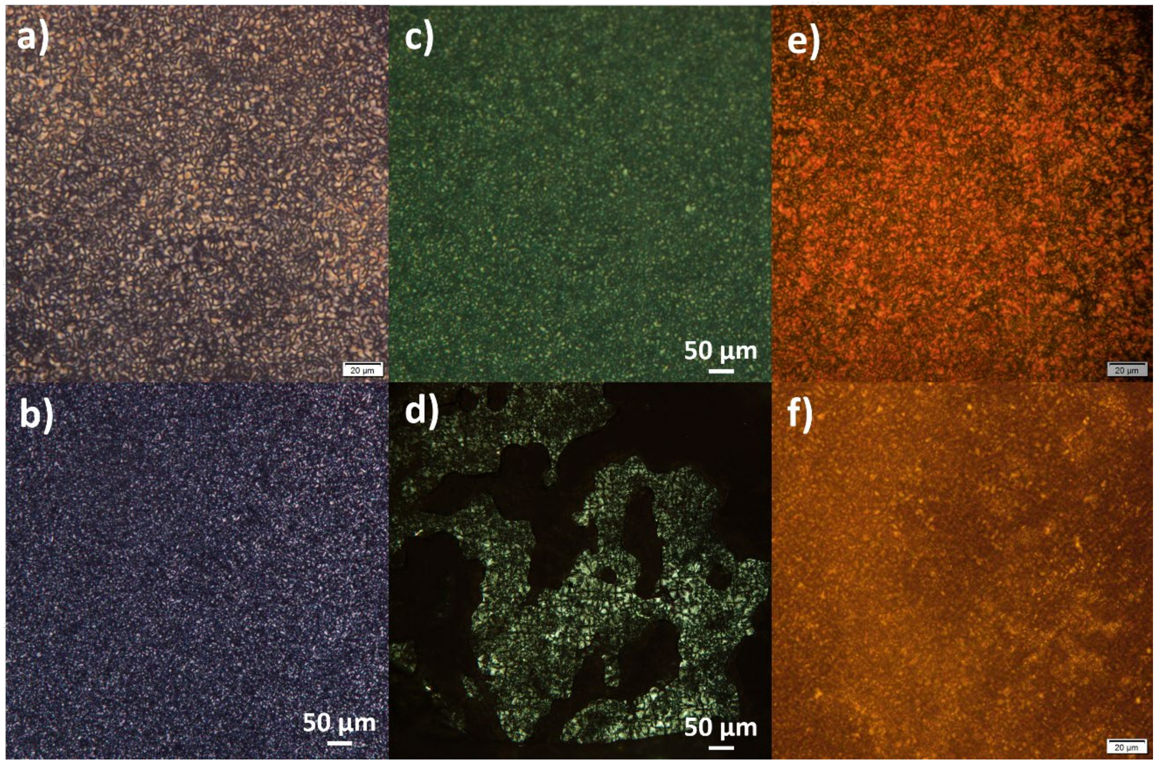

Figure S15. *POM pictures in the cooling process of a)* ***B1 4-8-P5N10*** *at 85°C, b)* ***B1 10-8-P5N10*** *at 130°C, c)* ***Bi 4-8-P5N10*** *at 140°C, d)* ***Bi 10-8-P5N10*** *at 110°C, e)* ***Bazo 4-8 P5N10*** *at 169°C and f)* ***Bazo 10-8-P5N10*** *at 125°C.*

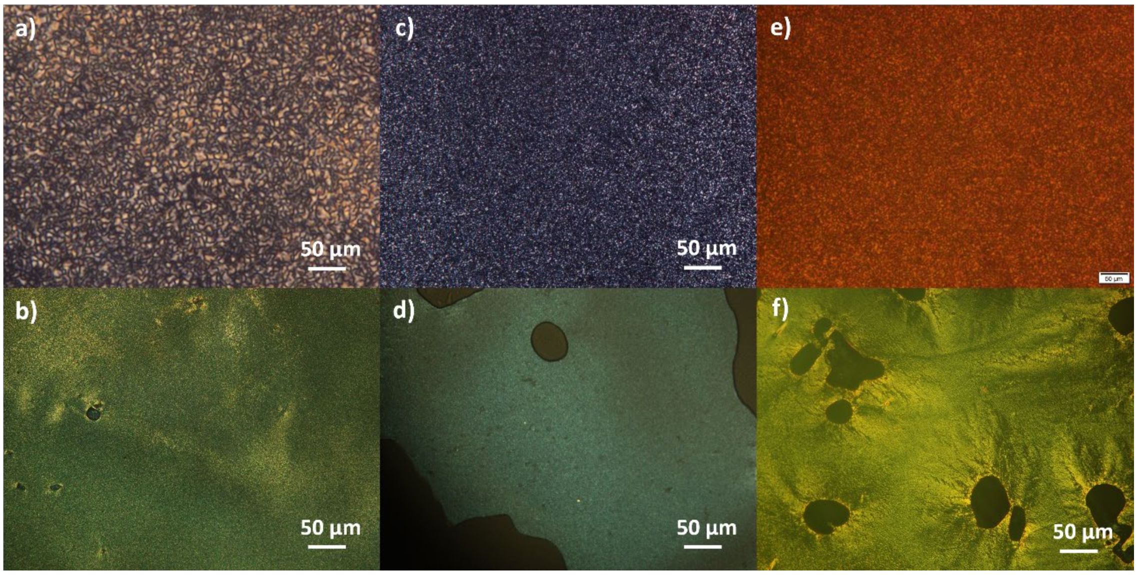

Figure S16. *POM pictures in the cooling process at room temperature of* ***B1 4-8-P5N10*** *a) in bulk and b) in the EIS cell,* ***Bi 4-8-P5N10*** *c) in bulk and d) in the EIS cell and* ***Bazo 4-8-P5N10*** *e) in bulk and f) in the EIS cell.*

3.4 DSC thermograms

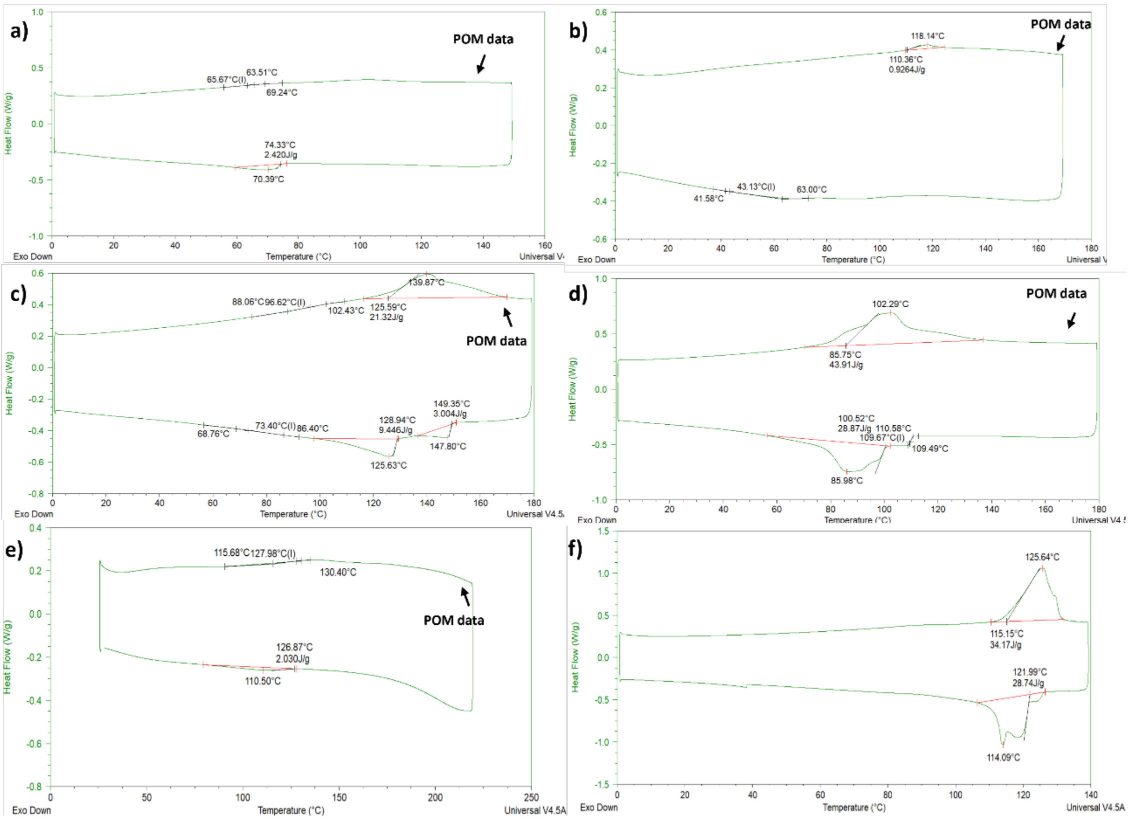

Figure S17. *2nd Heating-Cooling cycle at a rate of 10oC/min of a)* ***B1 4-8-P5N10****, b)* ***B1 10-8-P5N10****, c)* ***Bi 4-8-P5N10****, d)* ***Bi 10-8-P5N10****, e)* ***Bazo 4-8-P5N10*** *and f)* ***Bazo 10-8-1P5N10.***

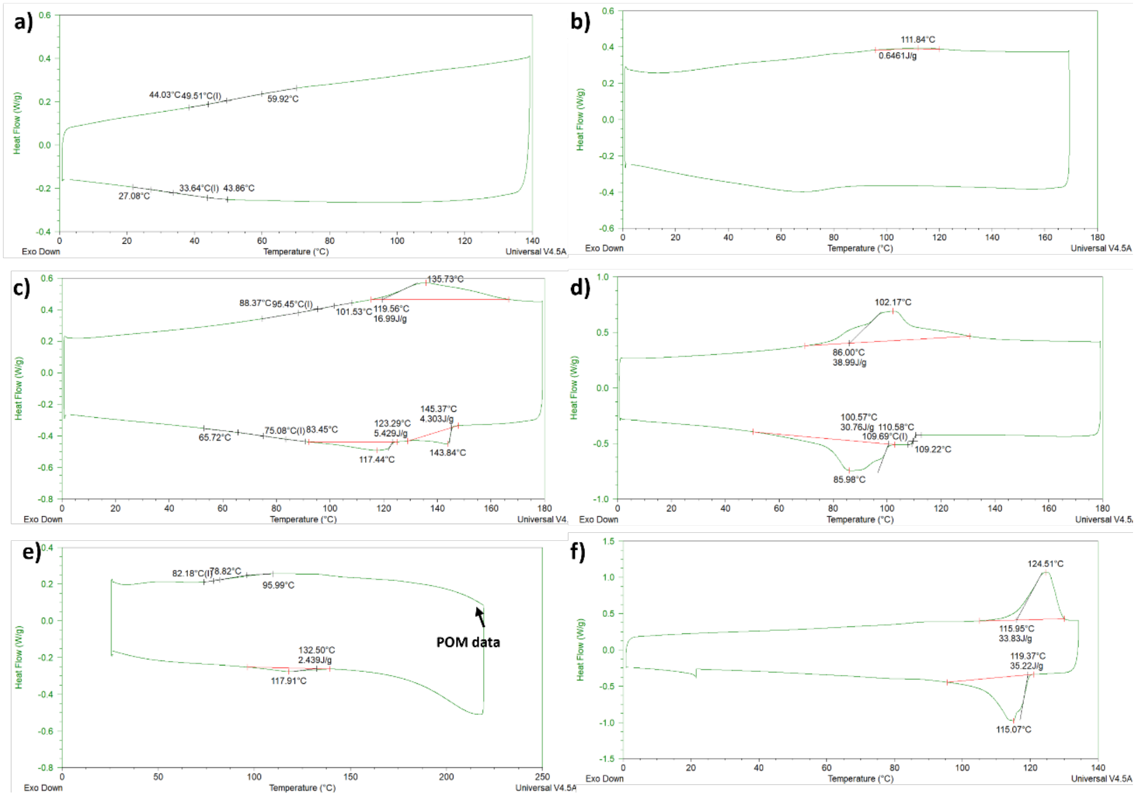

Figure S18*. 3rd Heating-Cooling cycle at a rate of 10oC/min of a)* ***B1 4-8-P5N10****, b)* ***B1 10-8-P5N10****, c)* ***Bi 4-8-P5N10****, d)* ***Bi 10-8-P5N10****, e)* ***Bazo 4-8-P5N10*** *and f)* ***Bazo 10-8-P5N10.***

3.5 X-ray diffractograms

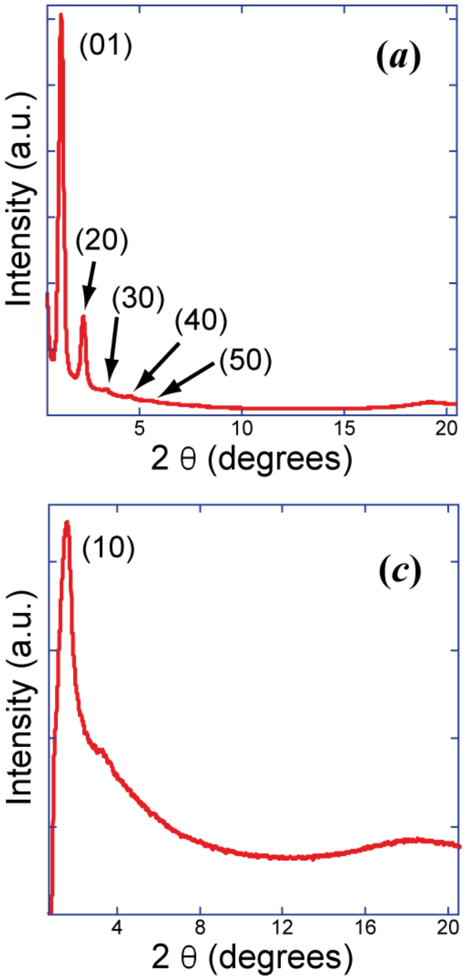

Figure S19. *X-Ray diffractograms (intensity represented in linear scale) of a)* ***Bi 4-8-P5N10*** *at 90°C, b)* ***Bi 10-8-P5N10*** *at 70°C, c)* ***Bazo 4-8-P5N10*** *at 200°C and d)* ***Bazo 10-8-P5N10*** *at 140°C.*

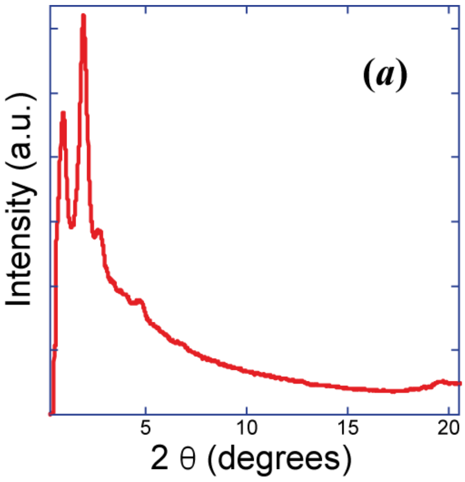

Figure S20. *XRD patterns (intensity represented in linear scale) of a)* ***Bazo 10-8-P5N10*** *at room temperature in the cooling cycle and b)* ***Bazo 10-8-P5N10*** *at 120°C in the cooling cycle.*

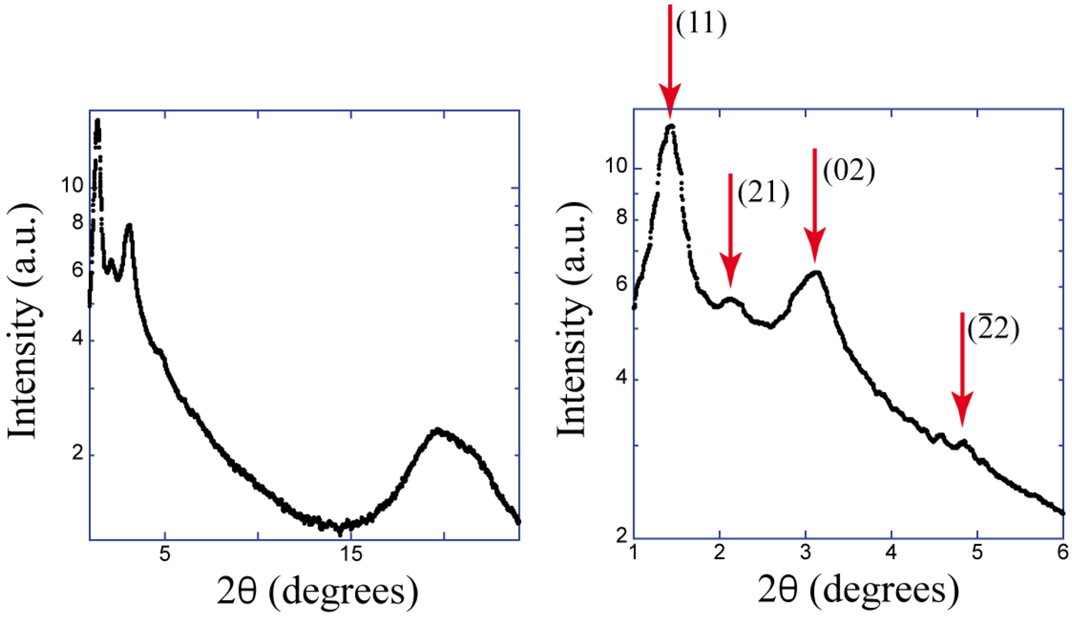

Figure S21. *XRD pattern of **B1 4-8-P5N10** at room temperature after filling the capillary in the isotropic phase and immediately cooled. The small angle region (right) shows a set of reflections that were indexed on the basis of an oblique lattice with parameters a = 81.4 Å, b = 64.8 Å, γ= 60.3º.*

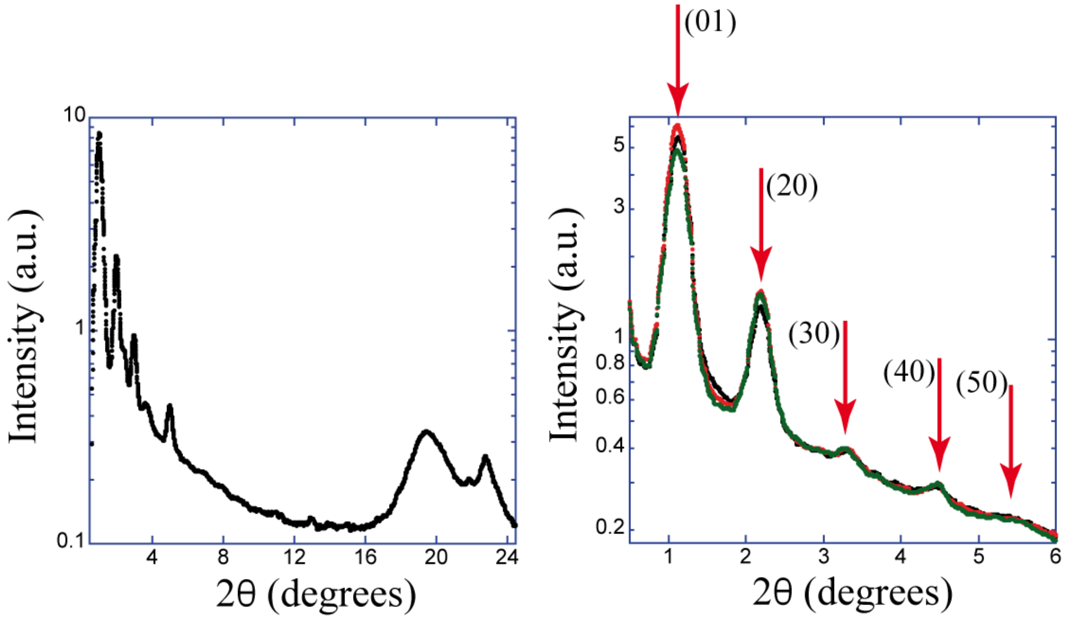

Figure S22. *Left: XRD pattern of **Bi 4-8-P5N10** at room temperature after filling the capillary in the isotropic phase and immediately cooled. Right: diagrams obtained during the cooling process from the isotropic phase (red 110ºC, black 90ºC, and green 60ºC).*

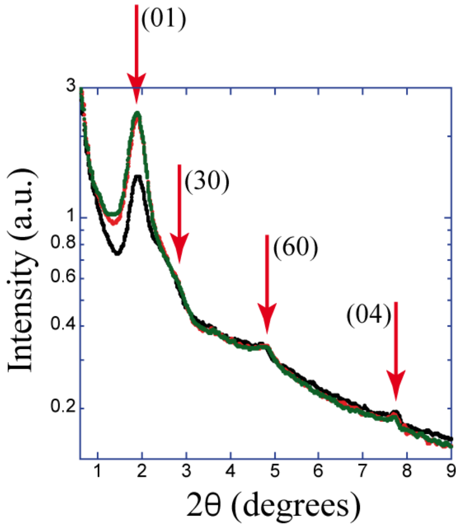

Figure S23. *XRD patterns of **Bi 10-8-P5N10** during the cooling process from the isotropic phase at several temperatures (black 90°C, green 70°C , red 50°C and purple 25°C).*

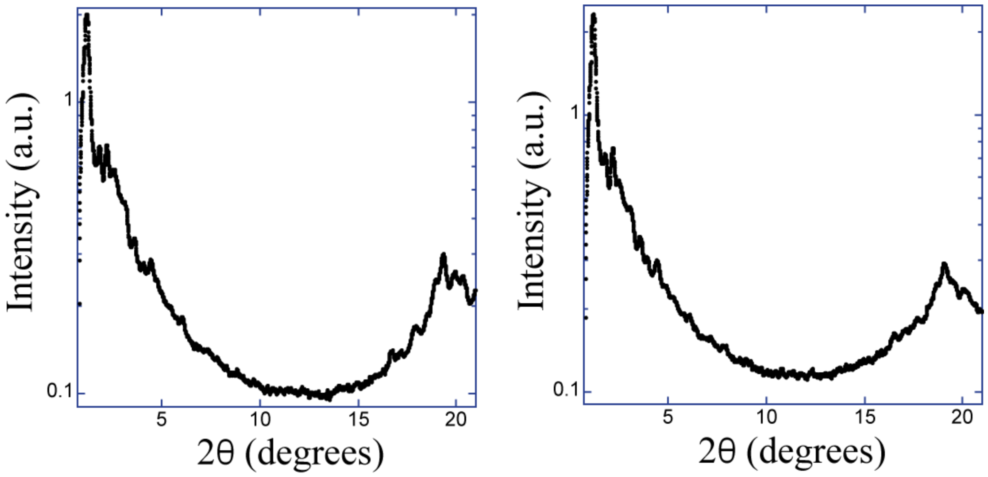

Figure S24. *XRD patterns of **Bazo 4-8-P5N10** at room temperature after filling the capillary in the isotropic phase and immediately cooled (left) and at 140°C (right) in the heating process. The material does not show a mesophase character until it is heated up to about 200°C (**Figure S19c**).*

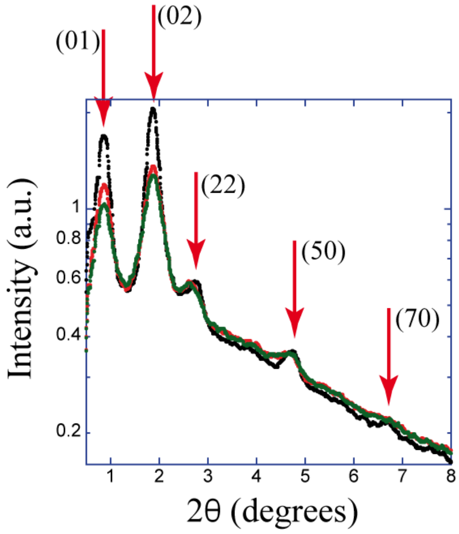

Figure S25. *XRD patterns of **Bazo 10-8-P5N10** at several temperatures on heating (black 140 °C), and on cooling from the isotropic phase (red 120°C, green 100°C).*

3.6 EIS measurements

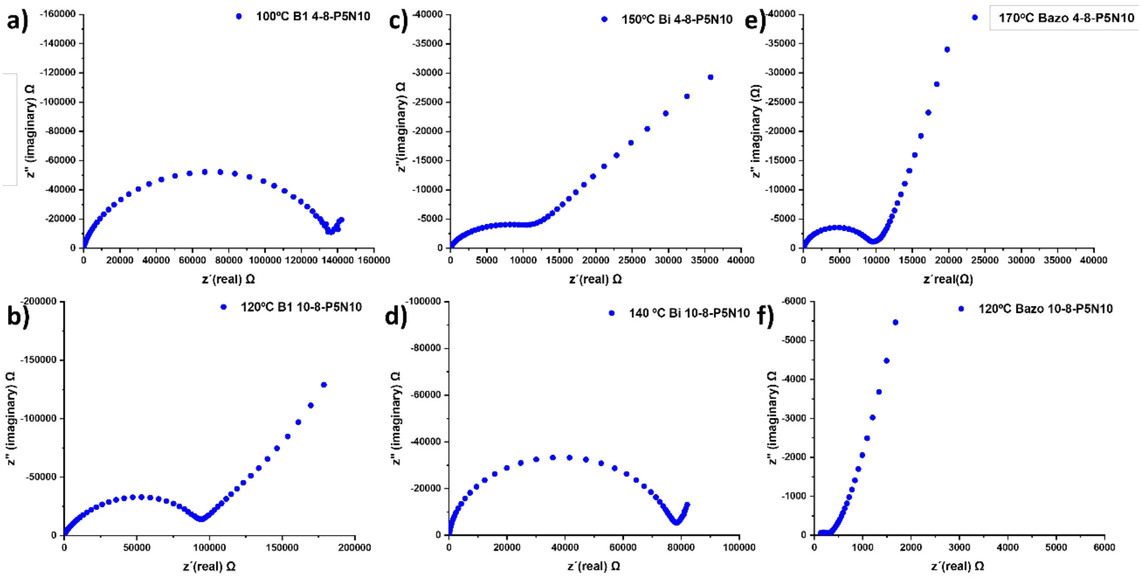

Figure S26. Nyquist plots of a) ***B1 4-8-P5N10*** *at 100ºC, b)* ***B1 10-8-P5N10*** *at 120ºC, c)* ***Bi 4-8-P5N10*** *at 150ºC, d)* ***Bi 10-8-P5N10*** *at 140ºC, e)* ***Bazo 4-8-P5N10*** *at 170ºC and f)* ***Bazo 10-8-P5N10*** *at 120ºC.*

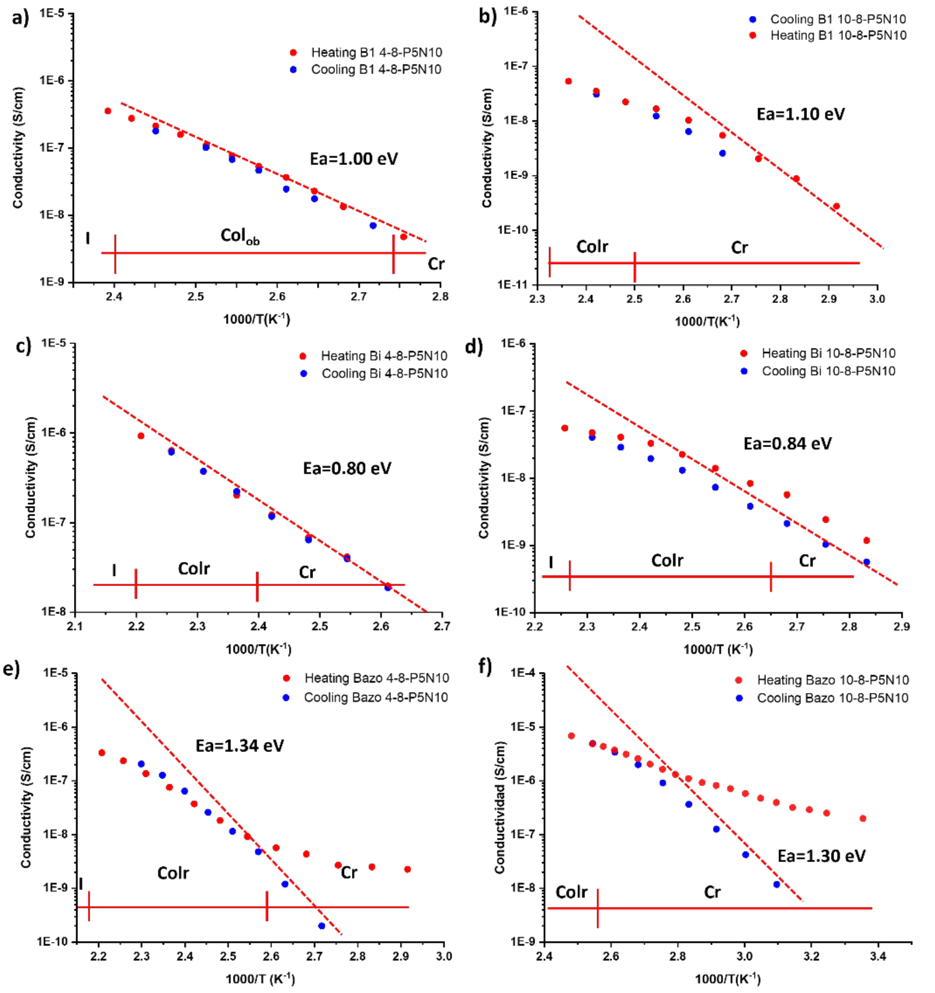

Figure S27. Conductivity variation in the heating-cooling process for the ionic bent-core pillar[5]arenes and activation energy (the straight lines with the indicated slopes are a guide to the eye).

3.7 UV-vis spectra

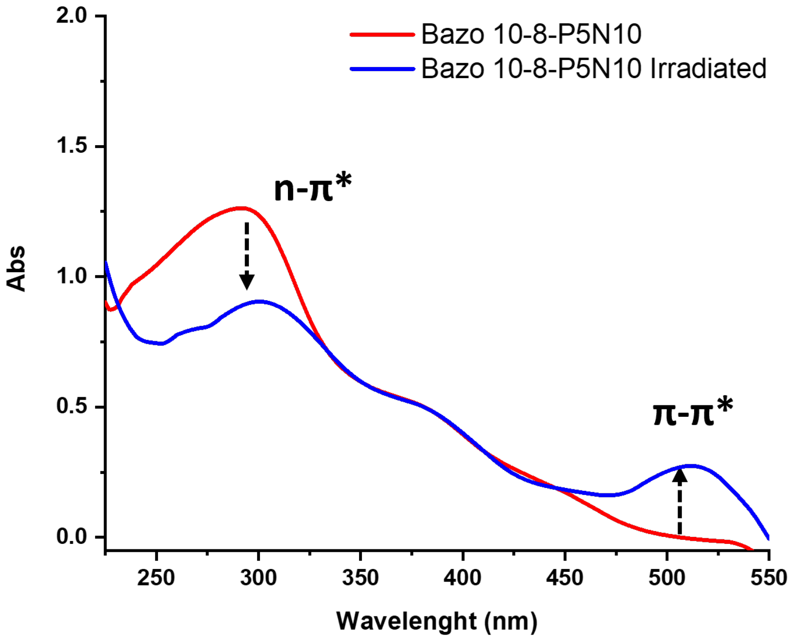

Figure S28. UV-vis spectra before (red line) and after (blue line) irradiation of the **Bazo 10-8-P5N10** in ITO-coated cell.

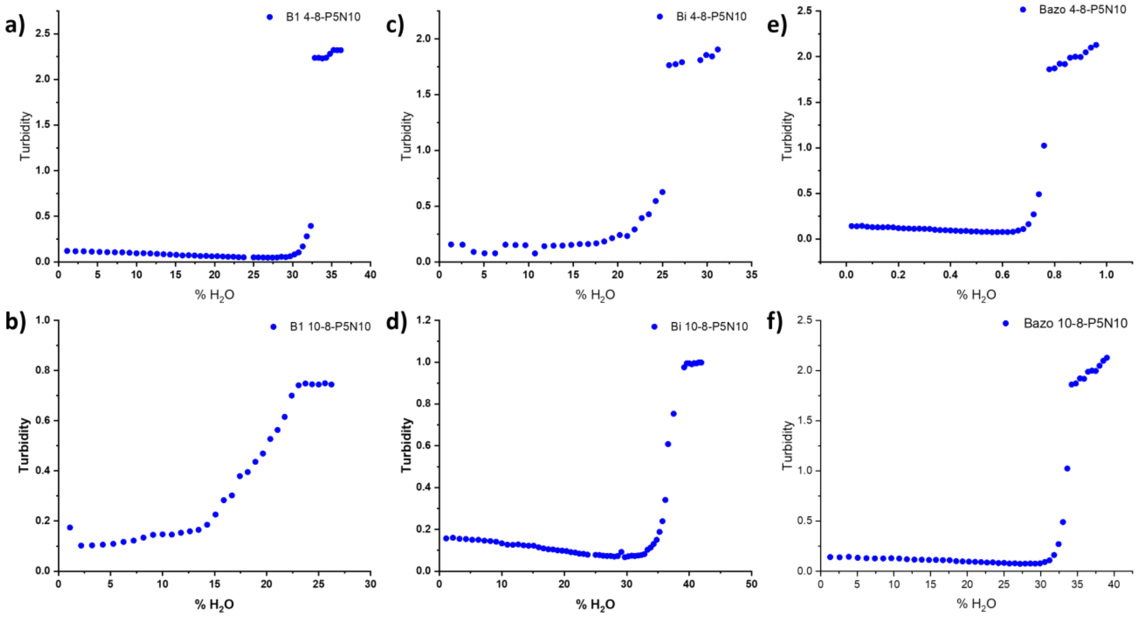

Figure S29. *Turbidity curves of a)* ***B1 4-8-P5N10****, b)* ***B1 10-8-P5N10****, c)* ***Bi 4-8-P5N10****, d)* ***Bi 10-8-P5N10****, e)* ***Bazo 4-8-P5N10*** *and f)* ***Bazo 10-8-P5N10****.*

3.8 TEM images

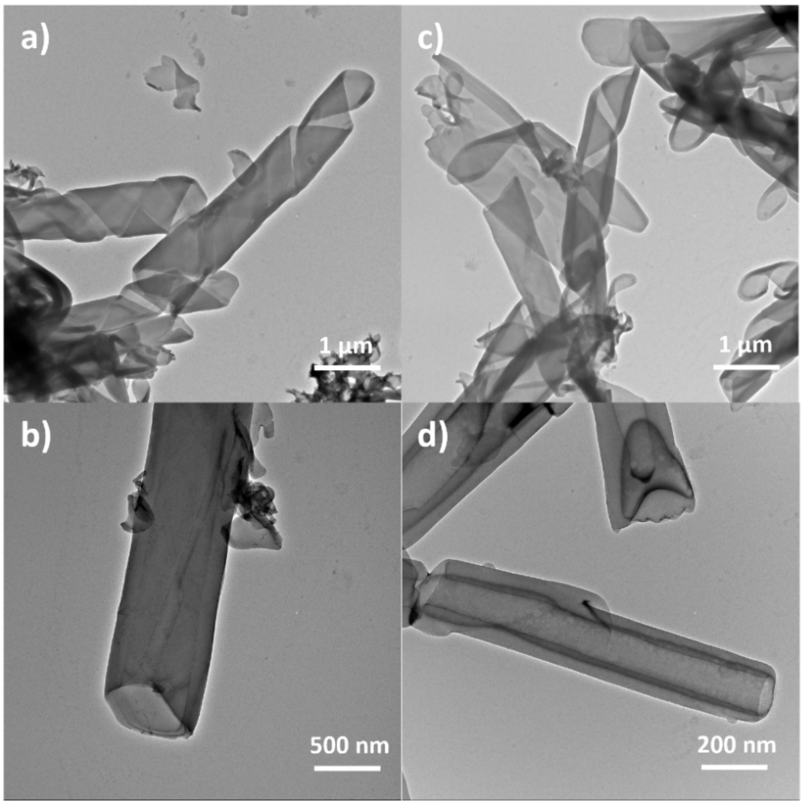

*Figure S30. TEM images of **B1 4-8-P5N10** a) before and b) after dialyzing and **B1 10-8-P5N10** c) before and d) after dialyzing.*

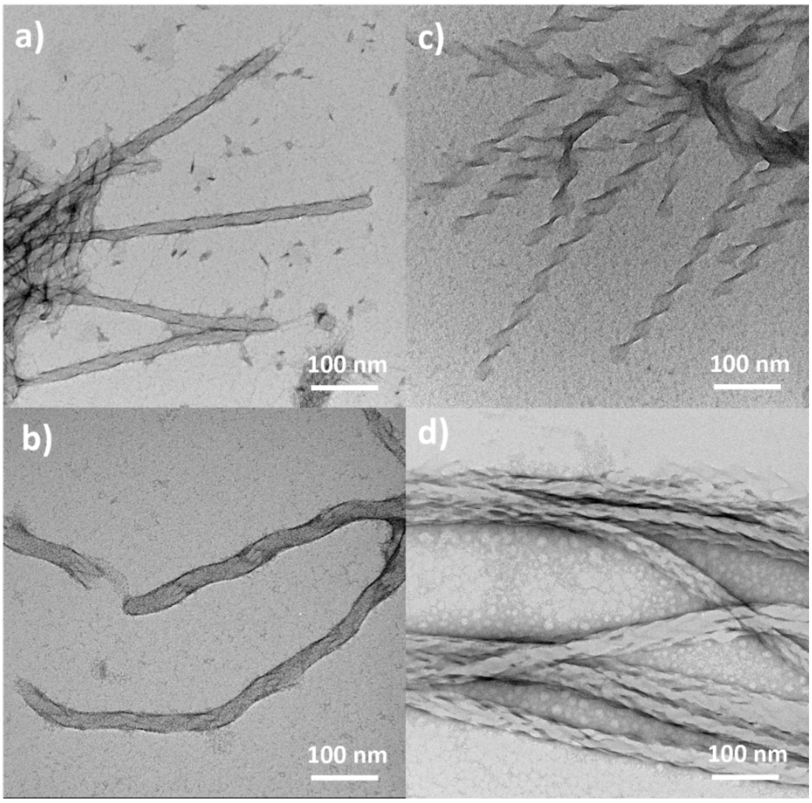

*Figure S31. TEM images of **Bi 4-8-P5N10** a) before and b) after dialyzing and **Bi 10-8-P5N10** c) before and d) after dialyzing.*

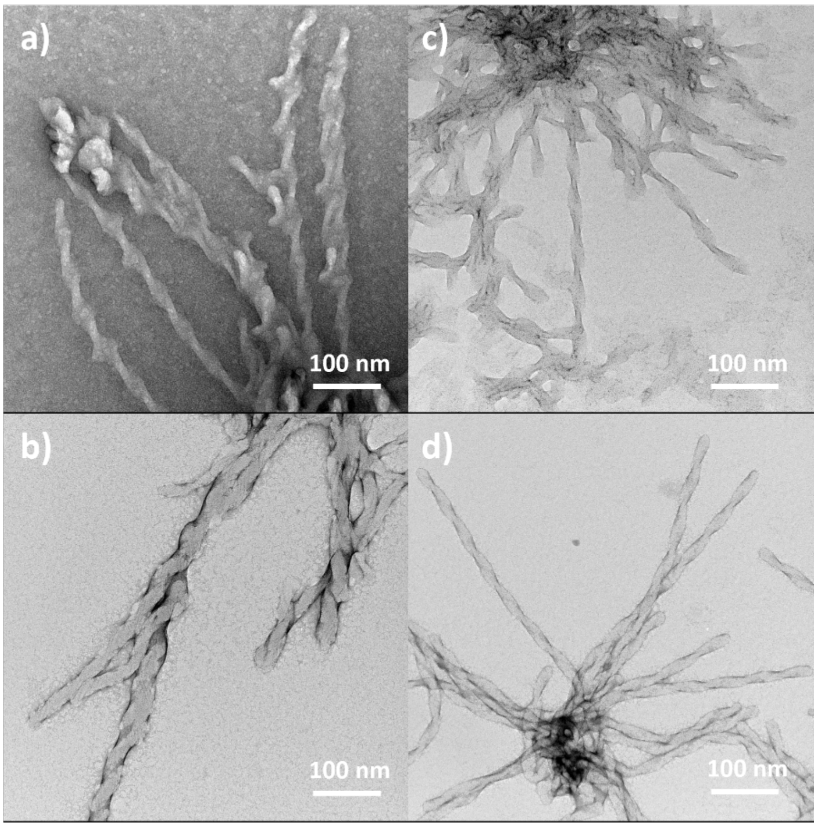

Figure S32. *TEM images of **Bazo 4-8-P5N10** a) before and b) after dialyzing and **Bazo 10-8-P5N10** c) before and d) after dialyzing.*
